# Optical Interactions and Excited-State Dynamics in γ‑Irradiated Mn-Doped Phosphate Glasses

**DOI:** 10.1021/acs.jpcb.6c02415

**Published:** 2026-06-25

**Authors:** José A. Jiménez

**Affiliations:** Center for Advanced Materials Science, Department of Biochemistry, Chemistry & Physics, 7604 Georgia Southern University, Statesboro, Georgia 30460, United States

## Abstract

The influence of gamma (γ)-irradiation (10 Mrad) on some physical and spectroscopic properties of phosphate glasses doped with MnO_2_ as 0.5, 2.0, 4.0, 6.0, and 8.0 mol % was assessed, ultimately focusing on the Mn^2+^ excited-state dynamics. A structural assessment by Fourier-transform infrared spectroscopy was overall consistent with the resilience of the glass structures to undergo significant alteration following γ-irradiation. However, the densities of the more heavily doped glasses (2–8 mol % MnO_2_) were somewhat higher after γ-irradiation, indicating a tendency toward more compact glasses. The absorption spectra of the pristine glasses exhibited a distinctive increase in the ^5^E_g_ → ^5^T_2g_ transitions band characteristic of Mn^3+^ (3d^4^) ions at high manganese content, which was greatly enhanced after γ-irradiation, consistent with Mn^2+^ photo-oxidation. Optical band gap energies were estimated and compared showing that the γ-irradiated glasses had lower values than the pristine as expected due to electron centers being trapped in the glass matrix. Photoluminescence (PL) spectroscopy showed the red emission from Mn^2+^ (3d^5^) ions was prominent in the pristine glasses, however with indications of a quenching effect above 4 mol % MnO_2_. The PL was then drastically quenched following γ-irradiation. The Mn^2+^ excited-state lifetimes ascribed to single and interacting ions were found to be consistently shorter for the γ-irradiated glasses despite the lowering in Mn^2+^ concentration expected given its photo-oxidation to Mn^3+^. Consequently, a Mn^2+^ → Mn^3+^ resonant energy transfer was proposed as a significant emission quenching pathway, which became enhanced after γ-irradiation.

## Graphical abstract

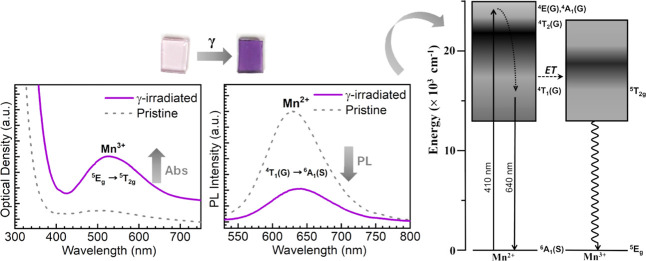

## Introduction

Manganese-containing glasses are versatile optical materials attractive for applications in light-emitting devices,^1,2^ radiation dosimetry,^3^ photochromic lenses,^4^ and solar spectral conversion.^5,6^ Within the different vitreous hosts, phosphate-based matrices are among the most desirable given their high transition metal solubility, low-melting character, and adequate properties achievable through compositional modifications.^1–4,6,7^ Hence, phosphate-based glasses continue to be the subject of interest for developing materials for photonic applications.^8–10^

An area of interest within the realm of Mn-doped phosphate glasses relates to studying the effects of ionizing radiation (e.g., gamma (γ)-irradiation) in connection with their potential use for switchable lenses and radiation dosimeters.^3,4^ Herein, acquiring a thorough understanding of the impact of the high-energy photons on material properties dictated by different valence states of manganese ions is crucial. Given the strong visible light absorption by Mn^3+^ and the intense red photoluminescence (PL) from Mn^2+^, evaluating the optical properties of the glasses is a common approach employed for assessing the impact of γ-irradiation.^7,12–14^ The remarkable PL of red-emitting Mn^2+^ ions further comprises their distinct decay dynamics, which can provide insights into the interactions with species in the solid host.^1,6,7,15^ However, an evaluation of Mn^2+^ emission decay kinetics is normally nonexistent in studies reporting on γ-irradiation effects on Mn-doped glasses.^4,7,12–14^ Information regarding the origin of the quenching of Mn^2+^ red emission in γ-irradiated glasses is currently limited, where a mere decrease in Mn^2+^ content following photo-oxidation to Mn^3+^ has been contemplated as a cause.^12^ Even though γ-irradiation effects on Mn-doped phosphate glasses have been previously reported, Mn^2+^ excited-state decay dynamics and associated energy transfer processes remain insufficiently understood. There is consequently a need for additional experimental work to bridge the existing knowledge gap and address the role of nonluminescent Mn^3+^ ions on Mn^2+^ PL quenching, which has been neglected. In this context, incorporating Mn^2+^ excited-state lifetime analysis within a spectroscopic investigation is considered valuable, and is as such a key novelty of the present work.

Typically, Mn-doped phosphate glasses studied in the literature have been subjected to accumulated doses of γ-irradiation within 6–10 Mrad.^7,12–14^ High doses are commonly deemed sufficient to reach saturation of spectral changes,^16^ wherein 100 kGy (10 Mrad) has been also considered an upper limit in radiation dosimetry experiments.^17^ Hence, in this work the γ-irradiation dose of 10 Mrad was chosen as a relatively high dose to study its effects on physical and optical properties of barium phosphate glasses doped with MnO_2_ as 0.5, 2.0, 4.0, 6.0, and 8.0 mol %. The pristine and γ-irradiated glasses were herein characterized regarding physical properties by Fourier-transform infrared (FT-IR) spectroscopy and density measurements. Absorption and PL spectroscopy measurements including the recording of emission decay curves were then conducted for a detailed assessment of the influence of γ-irradiation on optical properties. The entire evaluation leads to key physical insights into the origin of ionizing radiation-induced PL quenching generally applicable to red-emitting Mn^2+^-doped glasses having significant quantities of nonluminescent Mn^3+^ ions.

## Experimental Section

### Material Preparation and Processing

The glasses were synthesized by melting at 1150 °C in air atmosphere as described prior^15^ using as raw materials P_2_O_5_ (≥98%), BaCO_3_ (99.8%), and MnO_2_ (≥99.9). The glasses were made based on the 50P_2_O_5_:50BaO metaphosphate composition wherein different amounts of MnO_2_ were added in mol % in relation to network former P_2_O_5_. The glasses were doped with MnO_2_, adding it in quantities of 0.5, 2.0, 4.0, 6.0, and 8.0 mol %, and are labeled 05Mn, 2Mn, 4Mn, 6Mn and 8Mn, respectively. Following quenching on a steel mold, the glasses were annealed at 420 °C for 3 h to remove stress. The 05Mn glass appeared colorless whereas 2Mn was very similar; the remainder (4–8Mn) displayed a noticeable purple hue, which intensified with the increase in MnO_2_ content (photographs of the different glasses are presented in the inset of Figure 2a). This coloration is indicative of increasing amounts of Mn^3+^ ions arising from the thermal decomposition of MnO_2_, which also produces Mn^2+^ ions (e.g., 4MnO_2_ → 2Mn_2_O_3_ + O_2_↑ followed by 6Mn_2_O_3_ → 4Mn_3_O_4_ + O_2_↑).^15^

Samples of the 05–8Mn pristine glasses were subjected to γ-irradiation at room temperature (RT) to an accumulated dose of 10 Mrad (100 kGy) with a Co-60 source as in previous works.^18,19^ The γ-irradiated versions of the corresponding 05Mn, 2Mn, 4Mn, 6Mn, and 8Mn pristine glasses are herein referred to as 05Mn-Rad, 2Mn-Rad, 4Mn-Rad, 6Mn-Rad, and 8Mn-Rad. The glasses all exhibited a deep purple color, which intensified with MnO_2_ content; photographs of irradiated samples are shown in the inset of Figure 2b. All glasses were made as glass slabs about 1 mm thick for the optical measurements.

### Measurements

FT-IR spectroscopy was carried out at RT on the glasses as powders (crushed by mortar and pestle) with a Thermo Scientific Nicolet iS10 spectrometer equipped with an attenuated total reflectance (ATR) sampling accessory. The glass powders were pressed onto the ATR crystal and the measurements made consisting of 32 scans (4 cm^–1^ spectral resolution).

The densities of glass samples were measured at RT based on the Archimedes principle using a Mettler-Toledo XRS analytical balance equipped with a density measuring kit. The immersion liquid was distilled water, and the measurements were made in triplicate. The average densities obtained are reported herein along with the standard deviations.

Absorption spectra were collected at RT with an Agilent Cary 5000 double-beam spectrophotometer with a step size of 1 nm. The glasses were measured as the ∼1 mm thick slabs fixed on a sample holder for solids. The reference during measurements was air.

Steady-state PL emission and excitation spectra were recorded (1 nm step size) with a Horiba spectrofluorometer at RT using a continuous Xe lamp. A Xe flash lamp (∼2 μs pulses) operating at 10 Hz was further employed for obtaining emission decay curves with the excitation and emission wavelengths later specified.

## Results and Discussion

### FT-IR Spectroscopy

Phosphate glasses such as those studied herein made with up to 8 mol % MnO_2_ have been previously studied by X-ray diffraction and FT-IR spectroscopy besides optical properties in a comparative study of the effects of adding Si as reductant.^15^ The effectiveness of the glass synthesis procedure has been thus previously shown. This work then extends the FT-IR evaluation to compare the glasses before and after γ-irradiation and discusses results from density measurements prior to evaluating the optical properties in detail.

Figure 1a,b show the FT-IR spectra obtained for the pristine and γ-irradiated Mn-doped glasses, respectively. The spectra all bear resemblance before and after γ-irradiation exhibiting analogously the stretching vibrations characteristic of the phosphate network as seen for similar glasses.^7,12–15,19^ For instance, both the pristine 05Mn glass and the 05Mn-Rad counterpart show the broad band centered around 740 cm^–1^ (located within 650–800 cm^–1^) followed by the peak around 873 cm^–1^ ascribed to the symmetric and asymmetric stretching vibrations of P–O–P bridges in *Q* ^2^ units (PO_4_ tetrahedra with 2 bridging oxygens, BOs), respectively. Then the 05Mn and 05Mn-Rad glasses both exhibit in Figure 1a,b, respectively, the peak at about 1078 cm^–1^ credited to the asymmetric stretch of nonbridging oxygens (NBOs) in *Q* ^1^ units (PO_4_ tetrahedra with 1 BO) and the band around 1242 cm^–1^ associated with the asymmetric stretches of PO_2_^–^ groups in *Q* ^2^ units.

**Figure 1:**
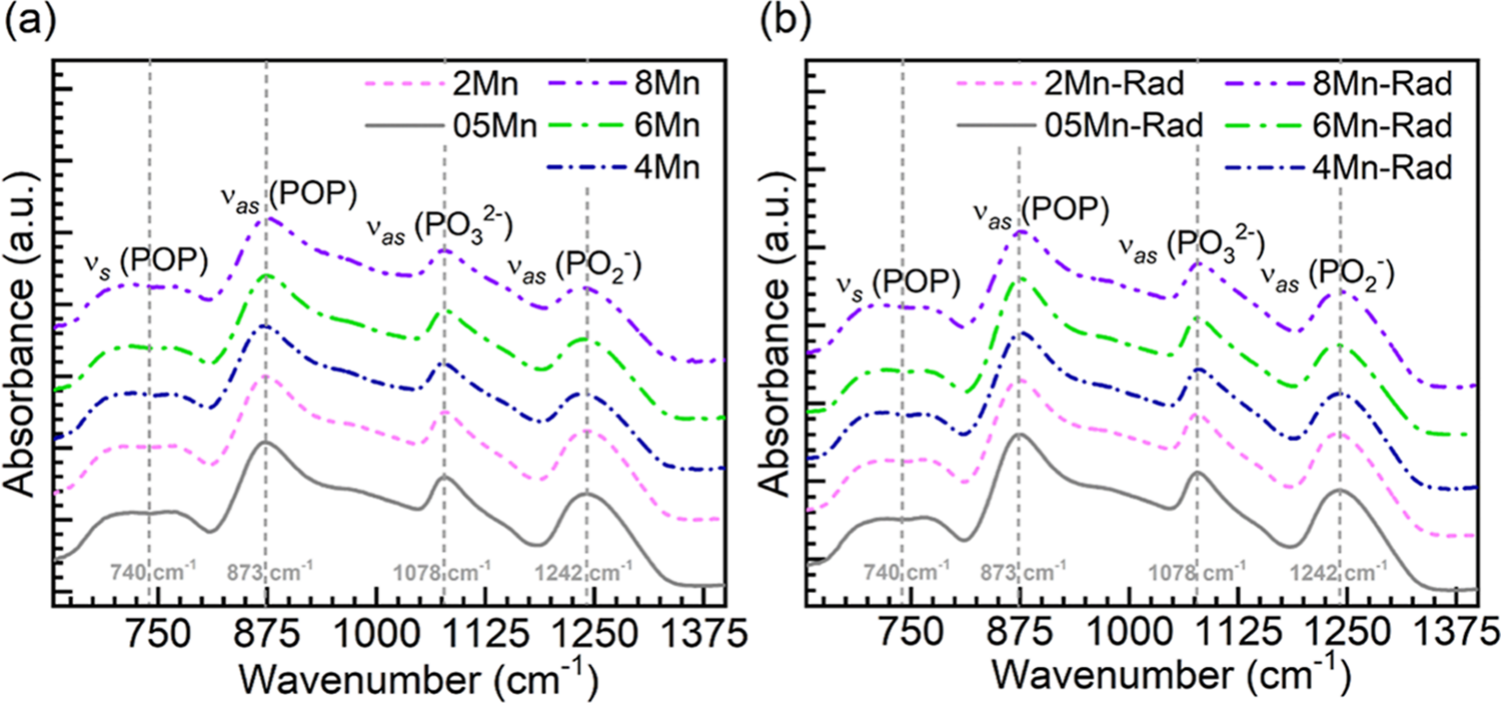
FT-IR spectra obtained for: (a) the pristine Mn-doped glasses and (b) the γ-irradiated counterparts.

The remainder of the spectra in Figure 1a,b concerning the pristine 2–8Mn glasses and their γ-irradiated counterparts appear upon direct visual comparison to not have any major spectral changes. However, as a more thorough approach to evaluate the impact of compositional changes or radiation processing on phosphate glasses, the FT-IR spectra may be further analyzed by deconvolution.^19–22^ Accordingly, Gaussian deconvolutions were herein performed to the FT-IR spectra of the pristine and γ-irradiated glasses in Figure 1a,b comprising seven bands as previously accomplished for similar glasses.^19,22^ The deconvoluted spectra are shown in the Supporting Information file in Figure S1 for the pristine 2–8Mn glasses and Figure S2 for the γ-irradiated counterparts. Tables S1 and S2 contain correspondingly the results pertaining to band position, full-width at half-maximum (fwhm) values, and assignments based on the literature.^19–22^ The broad band due to P–O–P symmetric stretches centered around 740 cm^–1^ was deconvoluted into pyrophosphate (P_2_O_7_^4–^) groups and metaphosphate chains at lower and higher wavenumbers, respectively. Besides the readily identified in Figure 1a,b, other components resulting from decomposing the spectra were the asymmetric P–O–P stretches of metaphosphate groups encompassing large and smaller rings such as the cyclotriphosphate (P_3_O_9_^3–^) and the symmetric stretch from PO_2_^–^ groups as identified in Tables S1 and S2.

Among the pristine 05–8Mn glasses, only minor variations with fluctuating values in the extracted parameters are observed in Table S1. At high manganese content, phosphate glasses have been shown by FT-IR to become progressively depolymerized.^23^ However, given the lack of substantial changes or clear trends in other features, a gradual evolution cannot be herein established for the 05–8Mn glasses. This implies the amounts of MnO_2_ employed in the present work are still relatively low and not sufficient to cause major structural changes on the glass network that can be followed by FT-IR. This is consistent with the previous study for the barium phosphate glasses made with up to 8 mol % MnO_2_, which also included Si as a reductant and showed no clear differences in the FT-IR spectra.^15^ With respect to the results for the γ-irradiated glasses in Table S2, the results similarly show fluctuating values with minor variations among the different glass samples. Overall comparison with the results for the pristine glasses in Table S1 also points to the small differences being associated with experimental variability. The deconvolution results as well as visual comparison of the spectra in Figure 1a,b then support the preservation of the structural features after γ-irradiation. This outcome is consistent with other experiments of barium phosphate glasses subjected to the same dose of 10 Mrad (100 kGy) of γ-irradiation wherein vibrational spectroscopy results indicated a resilience of the glass structures to undergo significant alteration after γ-irradiation.^18,19,24^

### Density Evaluation

The effect of γ-irradiation on the density of different glass systems has been studied by various authors;^25–28^ however reports on γ-irradiated Mn-doped phosphate glasses are scarce.^29^ Hence, in this work the density of the various glasses under consideration was evaluated for a comparison before and after γ-irradiation. The results are summarized in Table 1. Regarding the pristine 05–8Mn glasses the densities are observed to first increase up to 4Mn but then fluctuate and thus a definitive trend is not established. Comparing the pristine with the γ-irradiated counterparts, it is noticed that the 05Mn and 05Mn-Rad glasses have comparable densities within the estimated uncertainties. However, the densities for 2Mn-Rad, 4 Mn-Rad, 6Mn-Rad, and 8Mn-Rad all appeared distinctively higher than their pristine versions. It then seems that having relatively higher amounts of manganese doping favors a compaction effect upon γ-irradiation. Several authors have reported on the densification of various glasses subjected to γ-irradiation and linked the results with structural changes.^25,27,28^ On the contrary, for soda-lime phosphate glasses doped with 1 and 2 mol % MnO_2_, Hamdy et al.^29^ reported lower densities after exposing the glasses to γ-irradiation at a dose of 8 Mrad. The authors interpreted the results in terms of the formation of vacancies or breakdown of the glass network claimed to be supported by the FT-IR spectra.^29^ In the present case however the densities in Table 1 after the γ-irradiation tended to be higher than the pristine versions at high MnO_2_ content, whereas the analysis of the FT-IR spectra (vide supra) was not suggestive of major structural changes being induced by γ-irradiation. It is then herein hypothesized that the densification effect induced by γ-irradiation results from strong Coulombic attraction between photogenerated electrons and holes leading to more compact glasses. The significant formation of the radiation-induced centers thus becomes the point of focus in the optical spectroscopy analysis considered next.

**Table 1:** Densities Measured for the Pristine and γ-Irradiated Mn-Doped Glasses

glass	density (g/cm^3^)	glass	density (g/cm^3^)
05Mn	3.675 (±0.003)	05Mn-Rad	3.678 (±0.004)
2Mn	3.686 (±0.003)	2Mn-Rad	3.698 (±0.004)
4Mn	3.703 (±0.005)	4Mn-Rad	3.726 (±0.006)
6Mn	3.687 (±0.006)	6Mn-Rad	3.699 (±0.002)
8Mn	3.695 (±0.001)	8Mn-Rad	3.704 (±0.002)

### Optical Absorption Analysis

Figure 2a,b show the UV–vis absorption spectra of the pristine glasses and their γ-irradiated counterparts, respectively, along with photographs of samples. The spectrum for the 05Mn pristine glass in Figure 2a shows an indistinct baseline resembling an undoped glass host.^30^ This is expected for such a colorless glass containing a low concentration of manganese. However, the presence of Mn^2+^ is revealed through the characteristic PL (vide infra) even when there are no obvious absorption features. The 2Mn glass exhibits a weak feature around 500 nm, and in going from 4Mn to 8Mn, a purple hue develops wherein the spectra for such glasses in Figure 2a display a growing absorption band with maxima around 510 nm. The broad band emerging around 500–510 nm in the 2–8Mn glasses is characteristic of ^5^E_g_ → ^5^T_2g_ transitions in Mn^3+^(3d^4^) ions in octahedral coordination.^2,7,15,30^ The 4–8Mn glasses also exhibit weak features around 410 nm due to the ^6^A_1_(S) → ^4^E­(G),^4^A_1_(G) transitions in Mn^2+^(3d^5^) ions.^6,15,30^ These observations agree with the thermal decomposition of MnO_2_ in the melts leading to a mixture of Mn^3+^ and Mn^2+^ ions in the glasses as appraised in a related work.^15^

**Figure 2:**
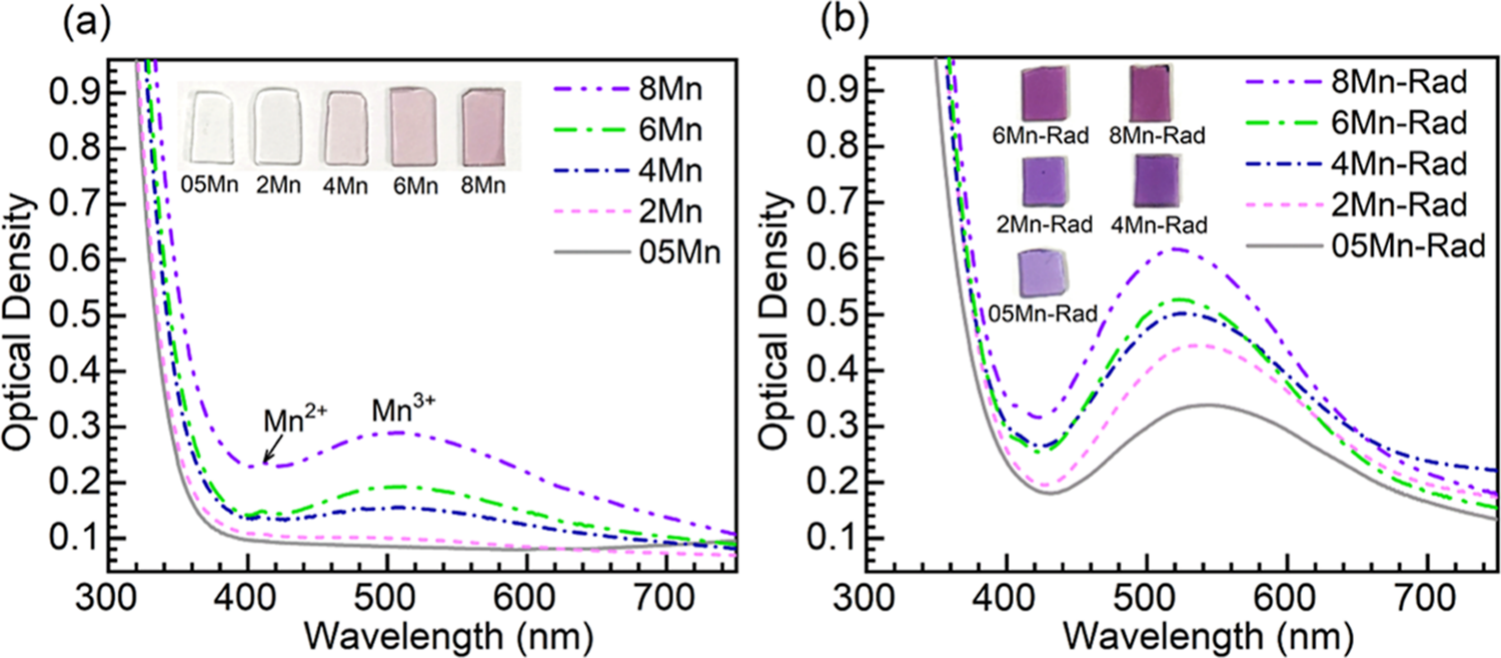
UV–vis absorption spectra recorded for: (a) the pristine Mn-doped glasses and (b) the γ-irradiated counterparts. The insets show photographs of samples for each of the glasses.

On the other hand, the γ-irradiated versions of the glasses all display in Figure 2b strong absorption in the visible and a purple color that appears to intensify with increasing manganese content. While the 05Mn glass showed no absorption features in Figure 2a, the spectrum for 05Mn-Rad in Figure 2b displays an intense band centered around 540 nm. The absorption band intensifies and its maximum undergoes a blue shift to about 535, 525, 523, and 520 nm for 2Mn-Rad, 4Mn-Rad, 6Mn-Rad, and 8Mn-Rad, respectively. Early work by Kreidl and Hensler^31^ on γ-irradiated Mn-doped phosphate glasses also showed that the photoinduced absorption band shifted toward shorter wavelengths with increasing γ-irradiation dose, which became more evident at higher MnO_2_ content. The behavior herein observed also resembles the progression seen with increasing manganese loading in phosphate glasses made with LiMn_2_O_4_ where the Mn^3+^ absorption band was first situated around 541 nm and shifted toward shorter wavelengths with increasing manganese content.^23^ Such an effect harmonizes with the increase in octahedrally coordinated Mn^3+^ ions in phosphate glasses.^12,32^

To better appreciate the differences produced by γ-irradiation, in Figure 3a–e the UV–vis absorption spectra of the pristine Mn-doped phosphate glasses are overlaid with their γ-irradiated counterparts. The radiation-induced bands are characteristic of the Mn^3+^ absorption as similarly appraised in previous works on γ-irradiated Mn-doped phosphate glasses.^4,7,12,31^ Analogous results were also reported by Möncke and Ehrt^33^ for Mn-doped phosphate glasses subjected to other types of radiation (e.g., 248 nm laser). In their study, the authors performed electron paramagnetic resonance (EPR) measurements but noticed that the evaluation of radiation-induced centers was not possible given the intense Mn^2+^ signal.^33^ The overwhelming effect of Mn^2+^ on EPR spectra of γ-irradiated Mn-doped phosphate glasses was also recognized by Ren et al.^4^ where the authors associated the weakening of the EPR signal with a decrease in Mn^2+^ concentration. It was acknowledged however by Möncke and Ehrt^33^ that the optical spectra presented the signs of photo-oxidized (Mn^2+^)^+^ with no observable phosphorus oxygen hole center (POHC) defects. The results herein are also indicative of Mn^3+^-like absorption rather than the formation of the POHC defects. POHC formation has been observed by optical absorption for other phosphate glasses in our group which have been exposed to the same γ-irradiation dose of 10 Mrad (100 kGy).^18,19,34^ Nevertheless, the POHC signature was not detected for glasses having elevated contents of iron^18^ and copper,^34^ pointing to a protective role by the transition metals at high concentrations. The current data thus indicate a shielding effect from manganese even at low levels, consistent with the FT-IR evaluation conducted (vide supra) supporting a lack of significant structural alteration. This concurs with Kreidl and Hensler^31^ recognizing in their early work that manganese acts as a “protecting” agent against γ-irradiation.

**Figure 3:**
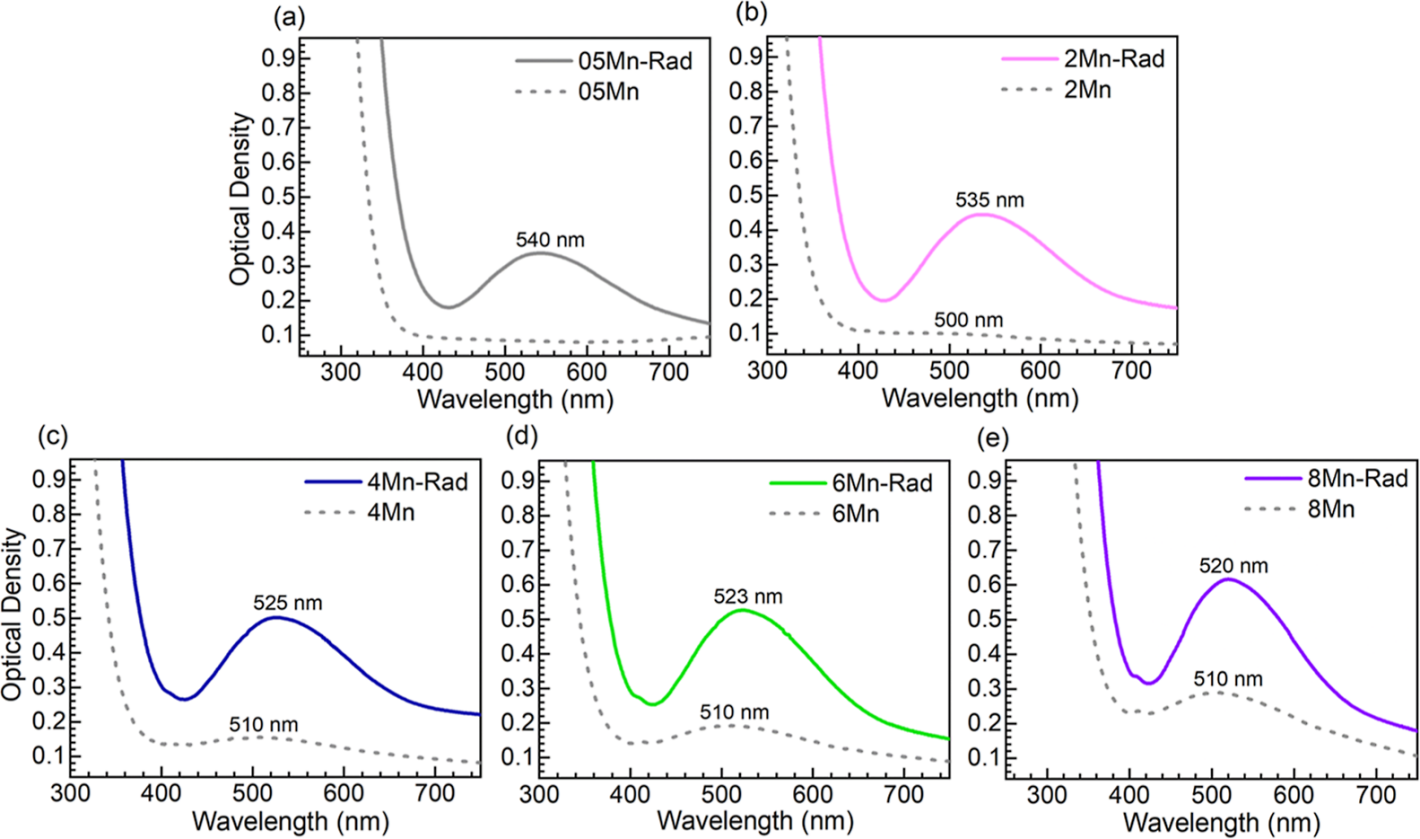
UV–vis absorption spectra of the pristine Mn-doped phosphate glasses (dotted curves) overlaid with their γ-irradiated counterparts (solid curves): (a) 05Mn and 05Mn-Rad; (b) 2Mn and 2Mn-Rad; (c) 4Mn and 4Mn-Rad; (d) 6Mn and 6Mn-Rad; and (e) 8Mn and 8Mn-Rad.

Admittedly, the formation of the Mn^3+^ color center in γ-irradiated phosphate glasses has been reported to become more pronounced at higher doses and increasing manganese content.^4,7,12,31^ The results obtained for the γ-irradiated samples herein are then consistent with the process of photo-oxidation of Mn^2+^ to Mn^3+^, which can be represented as

1 $${Mn}^{2+}\overset{\gamma }{\to }{Mn}^{3+}+EC$$

where electrons being ejected from Mn^2+^ form electron center (EC) defects. The photogenerated Mn^3+^ could be also represented^33^ as (Mn^2+^)^+^ given its inherent instability, which is reflected in that the process can be reversed by heat treatment.^4^ Regarding the nature of EC defects, iron impurities have been considered by Möncke and Ehrt^33^ as capable of capturing electrons and form the unstable (Fe^3+^)^−^ centers. Even if that was the case in the present work, having relatively high manganese concentrations implies that additional EC defects need to be produced to capture excess electrons. The Mn^3+^ color center formation may then involve the production of free electrons trapped by the glass matrix along with the holes being captured by the Mn^2+^ ions.^4^ The nature of radiation-induced EC defects in phosphate glasses has been postulated to be phosphorus-related in connection with PO_3_, PO_4_, and PO_2_ defects, which presumably absorb UV light at 210, 240, and 265 nm, respectively.^35^ From these, the occurrence of the PO_2_ EC defect has been linked to a distinct PL detected upon exciting γ-irradiated glasses at 265, which was observed whenever the POHC defects were evident.^18,19,34^ However, such type of radio-PL was not detected in the present work, which concurs with the lack of POHC signatures, and the protective role of manganese^31^ similarly realized with high concentrations of iron^18^ and copper.^34^ Henceforth, although the nature of the EC defects in the glasses under study is not clear, the evaluation continues focusing on their impact on optical band gap energies.

For evaluating the optical band gaps from UV–vis spectra, the absorption coefficient (α) is first obtained as

2 α=2.303×O.D./l

where O.D. is the optical density or absorbance and *l* is the sample thickness in cm. The data are then analyzed in the context of Tauc plots^19,30,36^ according to the expression for the absorption coefficient

3 $$\alpha =K\frac{{\left(h\nu -E_{opt}\right)}^{n}}{h\nu }$$

where *h* is Planck’s constant, ν is the photon frequency, *K* is a constant, the exponent *n* relates to the nature of the transitions (*n* = 2 for allowed-indirect transitions in glass), and *E*_opt_ is the optical band gap energy.^14,15,19,30^ A plot of (*E*α)^1/2^ vs photon energy (*h*ν) is then used to estimate *E*_opt_ from extrapolation of the linear portion of the plot and finding the intercept on the *x*-axis. The plots generated for the pristine glasses and γ-irradiated counterparts are presented in Figure 4a,b, respectively. The *E*_opt_ values estimated are listed in Table 2 along with the uncertainties stemming from the linear regressions performed yielding coefficient of determination values *R* ^2^ ≥ 0.995. Shown in Figure 5 is a plot of the band gap energies determined as a function of MnO_2_ added to the glasses for a comparison of the pristine with the γ-irradiated glasses. A decreasing trend is observed for the 05–8Mn pristine glasses with increasing MnO_2_ added, as similarly observed with increasing manganese loading in phosphate glasses made with LiMn_2_O_4_.^23^ Hence, this effect is prompted by the increase in the concentration of manganese ions and is likely due to an overwhelming effect from O^2–^ → Mn^2+^ and O^2–^ → Mn^3+^ charge transfer transitions.^37^ Further, the γ-irradiated Mn-doped glasses show distinctly (Table 2, Figure 5) lower *E*_opt_ values compared to the pristine samples. This outcome suggests that the EC defects produced by the ionizing γ-ray exposure alter the band gap through trapping an increased density of unpaired electrons below the bottom of the conduction band.^34,38,39^ The lowering of the band gap energies appears to be more pronounced for lower MnO_2_ concentrations, suggesting inhomogeneous electron density accumulation in the glass matrix. The band gap energies for the 05–8Mn-Rad glasses (Table 2, Figure 5) are however similar among themselves, especially considering the uncertainties in the determinations. This outcome seems related to the fact that the γ-irradiation dose was kept fixed for all glasses at 10 Mrad. This interpretation harmonizes with the dose-dependent formation of the color centers reported by Ren et al.^4^ for Mn^2+^ activated borophosphate glasses.

**Figure 4:**
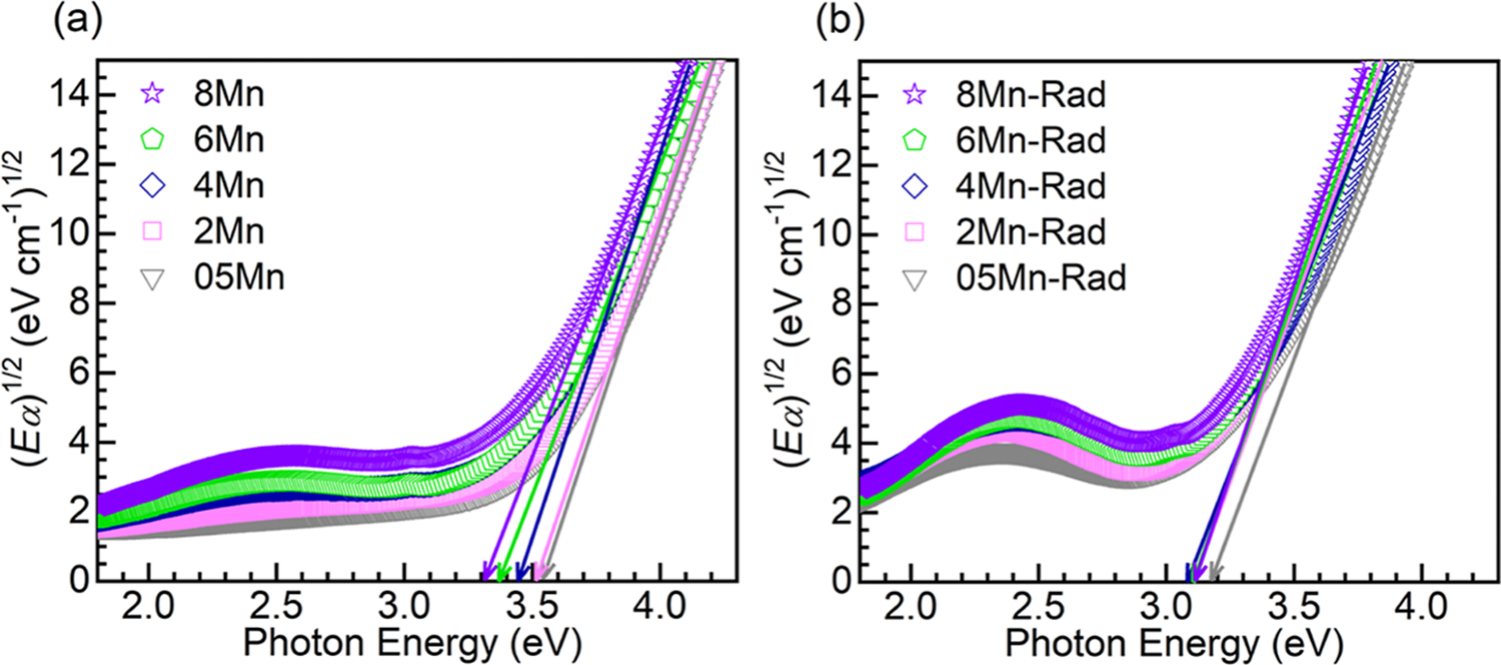
Tauc plots (indirect) for: (a) the pristine Mn-doped glasses and (b) the γ-irradiated counterparts. The lines with arrows represent the linear regressions and point to the estimated optical band gaps as the intercepts in the energy axis (values presented in Table 2).

**Table 2:** Indirect Optical Band Gap Energies (*E*_opt_) Estimated for the Pristine and γ-Irradiated Mn-Doped Glasses

glass	*E*_opt_ (eV)	glass	*E*_opt_ (eV)
05Mn	3.54 (±0.07)	05Mn-Rad	3.18 (±0.05)
2Mn	3.51 (±0.06)	2Mn-Rad	3.11 (±0.05)
4Mn	3.44 (±0.06)	4Mn-Rad	3.08 (±0.05)
6Mn	3.38 (±0.05)	6Mn-Rad	3.11 (±0.05)
8Mn	3.31 (±0.05)	8Mn-Rad	3.12 (±0.06)

**Figure 5:**
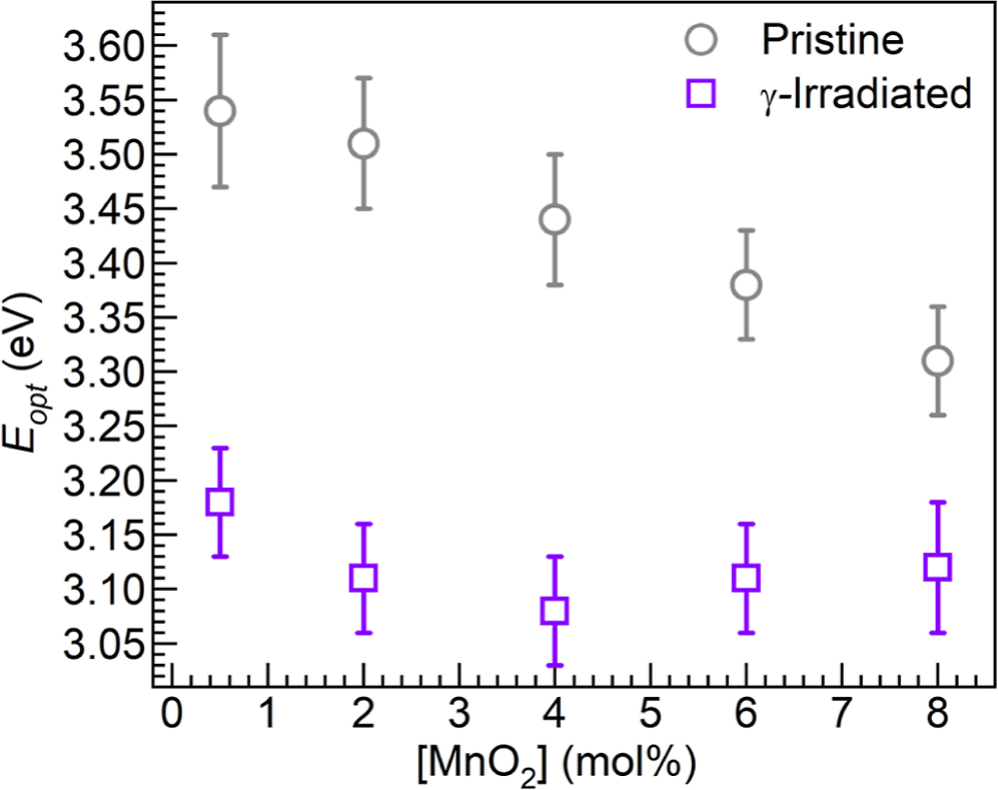
Plot of optical band gap energies (*E*_opt_) determined for the pristine and γ-irradiated glasses as a function of MnO_2_ added to the glasses (Table 2).

### Steady-State Photoluminescence Spectroscopy

Presented in Figure 6a are the steady-state PL excitation and emission spectra recorded for the glasses before γ-irradiation, i.e., 05Mn–8Mn. The emission spectra in Figure 6a were recorded under Mn^2+^ excitation of ^6^A_1_(S) → ^4^E­(G),^4^A_1_(G) transitions at 410 nm. They display the ^4^T_1_(G) → ^6^A_1_(S) transition^15^ emission band showing peaks at about 615, 620, 630, 638, and 638 nm for the 05Mn, 2Mn, 4Mn, 6Mn, and 8Mn glasses, respectively. Hence, these maxima were used as detection wavelengths when recording the PL excitation spectra also shown in Figure 6a, which show the typical peaks around 410 and 360 nm involving the ^6^A_1_(S) → ^4^E­(G),^4^A_1_(G) and ^6^A_1_(S) → ^4^T_2_(D) transitions in Mn^2+^, correspondingly.^1,40^ The 05Mn–8Mn glasses all exhibit considerable PL in Figure 6a, where the red shifts observed for the emission bands harmonize with an increase in the concentration of octahedrally coordinated Mn^2+^ ions.^1,7,15,40,41^ So even though the 05Mn and 2Mn glasses did not show any strong absorption features in Figure 2a, it is evident that they contain the red-emitting Mn^2+^ ions. Further, not only the emission peak position changes for the pristine glasses, but the PL excitation and emission spectra exhibit in Figure 6a a distinct evolution with respect to intensity. It is observed that the PL grows drastically from 05Mn to 4Mn, intensifies slightly for 6Mn, and ultimately appears similar for 8Mn compared to 6Mn. This type of behavior where the intensification of emission is suppressed points to a PL quenching effect being manifested at high manganese concentrations beyond the 4Mn glass.^11,23^ Particularly, it is noticed that the 6Mn and 8Mn glasses giving signs of concentration quenching also showed intensely in Figure 2a the absorption of nonluminescent Mn^3+^ ions. Thus, a potential pathway for the quenching of Mn^2+^ emission could be the Mn^2+^ → Mn^3+^ energy transfer argued for manganese-loaded phosphate glasses made with LiMn_2_O_4_.^23^ Additional information regarding the interaction of Mn^2+^ ions among themselves or with the Mn^3+^ color centers will be further considered in the light of emission decay dynamics data (vide infra).

**Figure 6:**
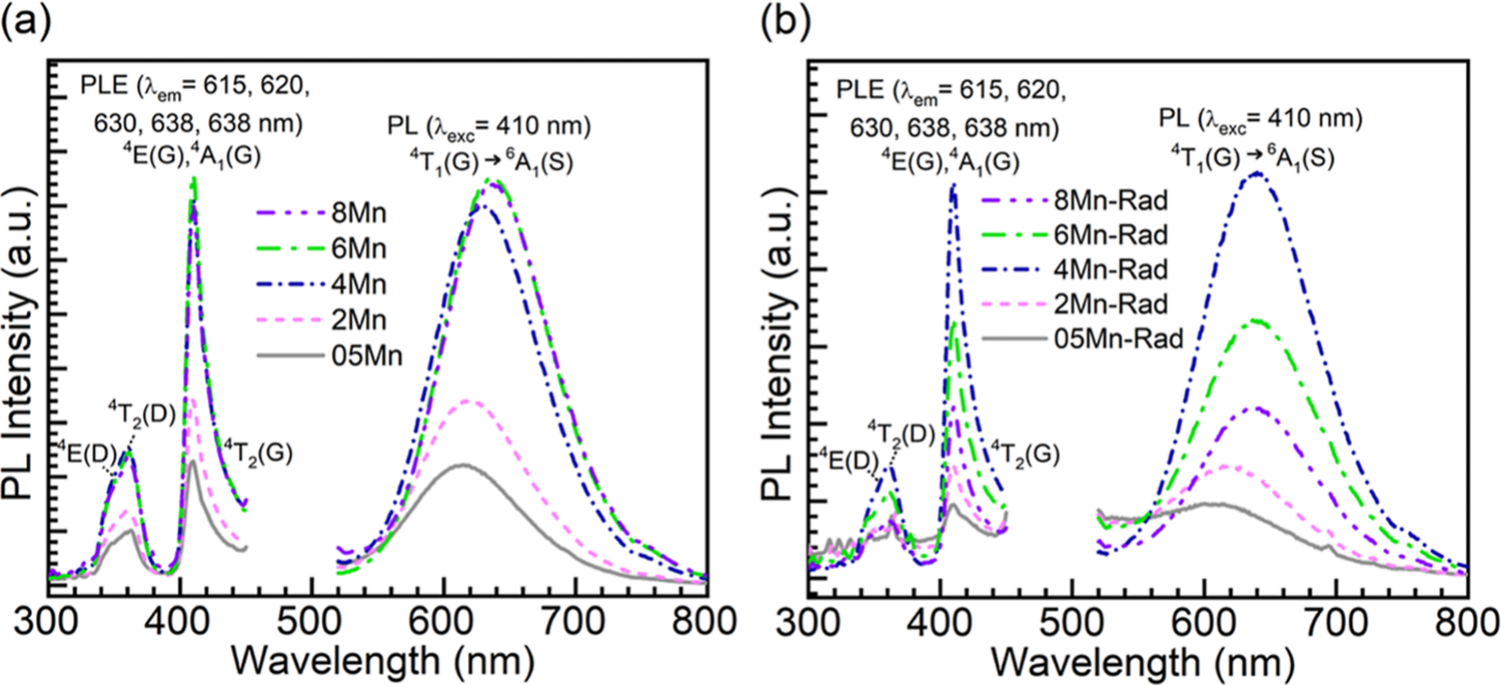
Excitation (PLE; excited states labeled) and emission (PL; ^4^T_1_(G) → ^6^A_1_(S) transition) spectra recorded for: (a) the pristine Mn-doped glasses and (b) the γ-irradiated counterparts. The PL spectra were all obtained under 410 nm excitation; the PLE spectra were recorded by monitoring emission at 615 nm for 05Mn and 05Mn-Rad, 620 nm for 2Mn and 2Mn-Rad, 630 nm for 4Mn and 4Mn-Rad, 638 nm for 6Mn and 6Mn-Rad, and 638 nm for 8Mn and 8Mn-Rad.

Figure 6b shows the PL excitation and emission spectra recorded for the γ-irradiated glasses. The conditions for recording the spectra were like those employed for the pristine glasses in Figure 6a. While the PL excitation spectra in Figure 6b only vary in intensity, the ^4^T_1_(G) → ^6^A_1_(S) red emission displayed maxima at about 610, 620, 640, 638, and 637 nm for the 05Mn-Rad, 2Mn-Rad, 4Mn-Rad, 6Mn-Rad, and 8Mn-Rad glasses, respectively. Similar progression of the emission peak wavelengths was observed for the pristine samples exhibiting in general a red shift with increasing manganese content (vide supra). However, the evolution of the intensities for the spectra in Figure 6b is such that the increasing trend is sustained from 05Mn-Rad to 4Mn-Rad, whereas the intensities for 6Mn-Rad and 8Mn-Rad diminish relative to 4Mn-Rad. Hence, following γ-irradiation, the glass with 4 mol % of MnO_2_ clearly shows a turning point in the PL output. This reflects an increased severity of the quenching effect taking place for high MnO_2_ concentrations after γ-irradiation.

The light-emitting behavior of the Mn-doped glasses may be further characterized via Commission Internationale de l’Eclairage (CIE) 1931 chromaticity diagrams.^2,6^ Based on the emission spectra in Figure 6a,b, the CIE diagrams with chromaticity results shown in Figure 7a,b were obtained pertaining to the pristine and γ-irradiated glasses, respectively. The (*x*, *y*) chromaticity coordinates obtained are summarized in Table 3. Also presented in Table 3 are the related color correlated temperatures (CCT) calculated from the following equation^42^

4 $$CCT=-449m^{3}+3525m^{2}-6823.3m+5520.33$$

where $m=\frac{\left(x-0.3320\right)}{\left(y-0.1858\right)}$ and (*x*, *y*) are the chromaticity coordinates. The red-emitting character of the pristine glasses clearly improves with increasing manganese content up to the 4Mn glass also having the lowest CCT of 1641 K (Table 3). Subtle changes are seen afterward for the (*x*, *y*) chromaticity coordinates of 6Mn and 8Mn as perceived from Figure 7a and Table 3. From these most concentrated pristine glasses, 6Mn displays the highest CCT of 1782 K, which approaches the 05Mn glass exhibiting utmost “warm” light with CCT at 1790 K. Applications wise, the 2Mn glass however appears most appropriate given the balance between luminescent and absorption properties as it did not display the Mn^3+^ band prominently in Figure 2a. Concerning the γ-irradiated glasses, Figure 7b and Table 3 show the (*x*, *y*) chromaticity coordinates and CCT values are more spread apart. Herein, the 05Mn-Rad and 2Mn-Rad samples displayed a shift toward more orange character with the highest CCT being obtained for 05Mn-Rad at 2727 K. The 4Mn-Rad glass, which exhibited the maximum PL in Figure 6b, then exhibited similar chromaticity coordinates and CCT results to its pristine counterpart 4Mn as seen from Table 3. The 6Mn-Rad and 8Mn-Rad glasses then show more noticeable differences in Table 3 compared to the 6Mn and 8Mn samples. It appears that the radiation-induced PL quenching impacts the results, thus warranting a PL comparison between pristine and γ-irradiated glasses as considered below.

**Figure 7:**
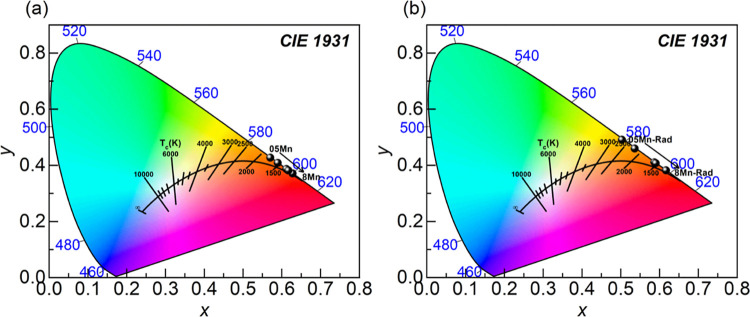
CIE 1931 chromaticity diagram with coordinates (sphere symbols) of emission color calculated for: (a) the pristine Mn-doped glasses and (b) the γ-irradiated counterparts from the emission spectra. The chromaticity coordinates (*x*, *y*) and CCT are summarized in Table 3.

**Table 3:** CIE 1931 Chromaticity Coordinates (*x*, *y*), and CCT Obtained for the Pristine Mn-Doped Glasses and Their γ-Irradiated Counterparts

glass	*x*	*y*	CCT (K)	glass	*x*	*y*	CCT (K)
05Mn	0.570	0.427	1790	05Mn-Rad	0.503	0.491	2727
2Mn	0.589	0.409	1650	2Mn-Rad	0.536	0.460	2212
4Mn	0.612	0.387	1641	4Mn-Rad	0.616	0.382	1668
6Mn	0.628	0.371	1782	6Mn-Rad	0.587	0.411	1662
8Mn	0.617	0.382	1671	8Mn-Rad	0.590	0.407	1646

To compare the PL of the γ-irradiated glasses with the pristine, presented in Figure 8a–e are the emission spectra overlaid for each glass as obtained under 410 nm excitation. It is evident that all γ-irradiated glasses experienced drastic quenching of the Mn^2+^ PL, which is consistent with the weakening of the Mn^2+^ emission reported for γ-irradiated phosphate glasses by several authors.^7,12,13^ Seeking a more quantitative appraisal, the emission spectra in Figure 8a–e were integrated and the obtained areas are plotted in Figure 9 as a function of MnO_2_ added to the glasses. While the progression of the integrated PL follows the prior discussion in connection with Figure 6a,b, respectively, it is noticed that the difference after γ-irradiation becomes more significant with manganese content. To further assess the increasing gap, the inset of Figure 9 plots the magnitude of the change in the integrated PL, ΔPL = |PL_γ‑Irradiated_ – PL_Pristine_|, as a function of MnO_2_ content. The graph clearly shows that the change in the integrated PL continuously increases throughout the glass set. However, the difference seems to increase to a lower extent after the 4Mn glass (see the solid line in the inset of Figure 9 intended as guide to the eye). Performing a linear regression to the data points yielded the equation, correlation coefficient (*r*), and coefficient of determination (*R* ^2^) displayed in the inset of Figure 9. The data show reasonable linearity, yet the correlation seems adversely affected by the lower degree of augmentation seen for 6Mn and 8Mn. Nevertheless, the evaluation indicates that in general the radiation-induced PL quenching becomes more pronounced as the concentration of MnO_2_ in the glasses increases. This appears to be linked to the increasing amounts of photogenerated Mn^3+^ color centers reflected in the increased absorption in Figure 2b, which becomes the subject of the forthcoming discussion.

**Figure 8:**
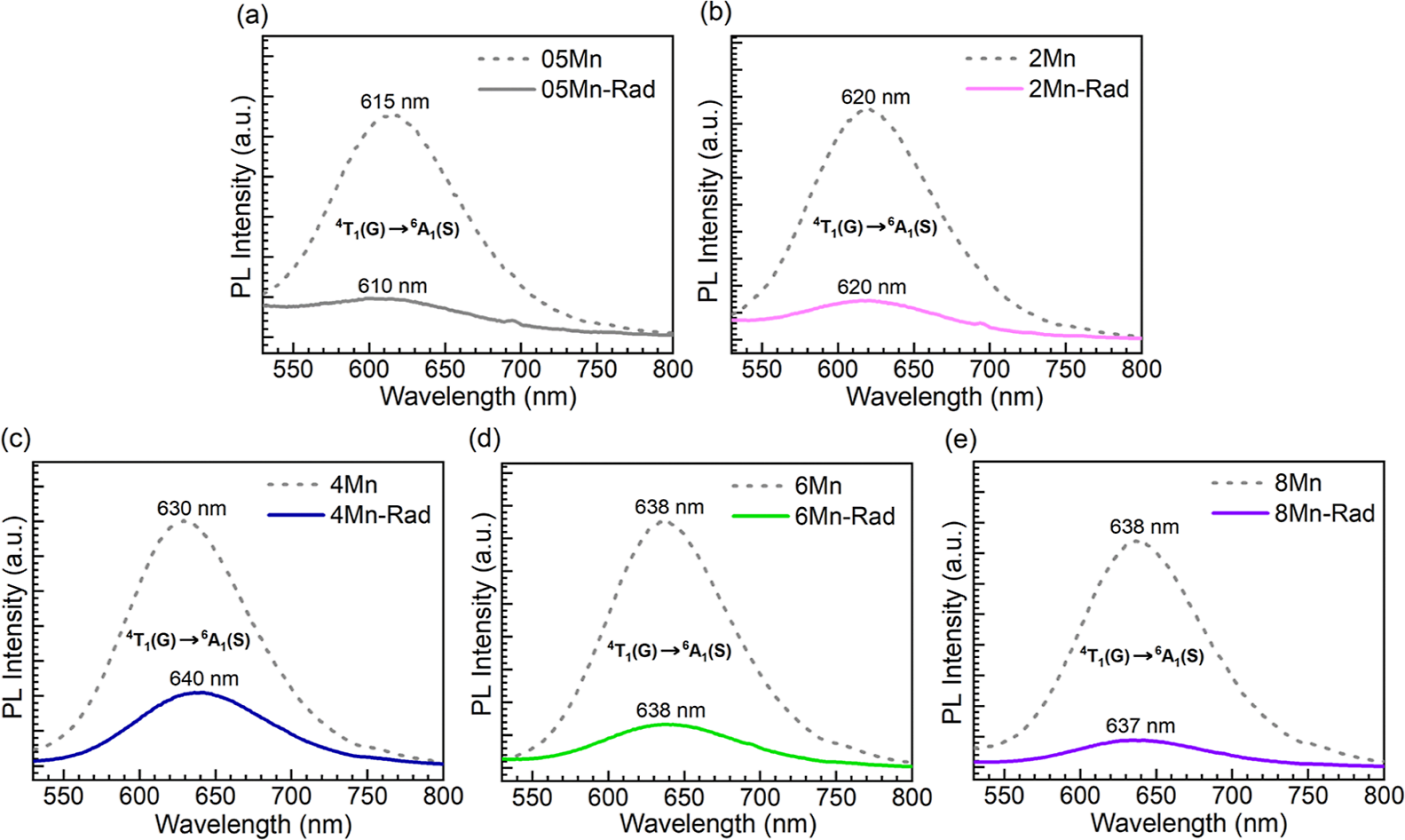
Emission spectra (λ_exc_ = 410 nm) of the pristine Mn-doped phosphate glasses (dotted curves) overlaid with their γ-irradiated counterparts (solid curves): (a) 05Mn and 05Mn-Rad; (b) 2Mn and 2Mn-Rad; (c) 4Mn and 4Mn-Rad; (d) 6Mn and 6Mn-Rad; and (e) 8Mn and 8Mn-Rad.

**Figure 9:**
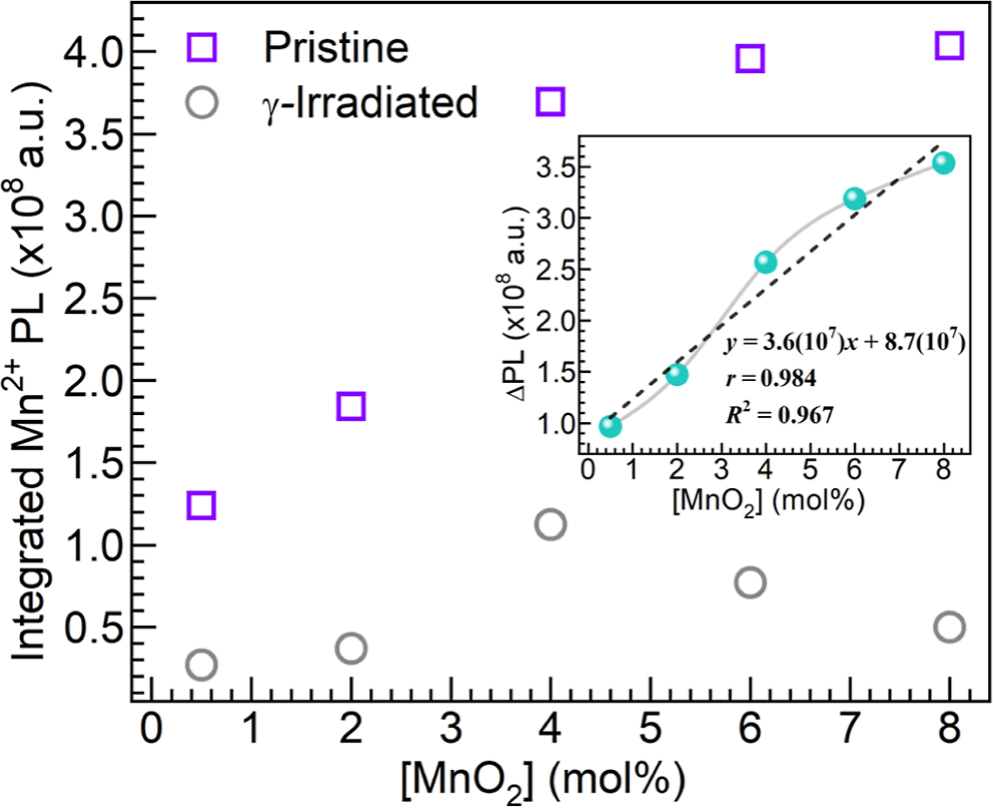
Plot of integrated PL intensities for the pristine Mn-doped phosphate glasses (open squares) and the γ-irradiated counterparts (open circles) as a function of MnO_2_ added to the glasses. The inset is a plot of the magnitude of the change in the integrated PL intensities (ΔPL) vs MnO_2_ content (the solid line is a guide to the eye); the dashed trace is a linear fit to the data (equation, correlation coefficient, *r*, and coefficient of determination, *R* ^2^, displayed).

A lowering of the Mn^2+^ concentration following the conversion into Mn^3+^ ions has been considered as the cause of the decreased Mn^2+^ emission after γ-ray exposure.^12^ This is likely a contributor in the present work as the absorption spectra considered in Figure 2b indicated that the photo-oxidation of Mn^2+^ to Mn^3+^ took place as represented by eq 1. However, a glass such as 8Mn would likely have a high concentration of Mn^2+^, facilitating concentration quenching via Mn^2+^–Mn^2+^ interactions.^6,11,30^ As so, a decrease of Mn^2+^ ions in such could be advantageous, or at least not as detrimental to the PL intensity as noticed from comparing Figure 6a,b, and in the overlay in Figure 8e. Hence, the possibility for Mn^3+^ ions to act as energy sinks more prominently than in the unirradiated glasses (e.g., 6Mn, 8Mn) may be considered to explain the overall behavior captured in Figure 9. In Figure 10, the PL spectrum of the 8Mn glass is overlaid with the absorption spectrum of the γ-irradiated version 8Mn-Rad, both in units of photon energy (eV). A considerable spectral overlap between Mn^2+^ emission and Mn^3+^ absorption is observed. This supports the likelihood of a resonance energy transfer from the Mn^2+^ ions as donors to Mn^3+^ ions as acceptors.^43,44^ In a situation where a nonradiative resonance energy transfer occurs, the energy is transmitted from the donor (D) to the acceptor (A) without photon emission. Hence, the luminescence of the donor originating in the state partaking in the transfer is quenched in the presence of the acceptor. The transfer rate (*W*_DA_) for a nonradiative energy transfer follows the expression given as

5 $$W_{DA}=\frac{2\pi }{\hbar }{\left|\int \Psi_{F}^{*}H_{1}\Psi_{I}\right|}^{2}\rho \left(E\right)$$

where Ψ_I_ and Ψ_F_ are the initial (D is excited while A is relaxed) and final (D is relaxed while A is excited) states, respectively, ρ­(*E*) is the density of states offered by vibrational motion, and *H*_1_ is the Hamiltonian for the Coulombic interaction that expanded produces the terms connected to the mechanisms of electric dipole–dipole, dipole–quadrupole, quadrupole–quadrupole, electric dipole–magnetic dipole and exchange interactions.^43,44^ Of the various possibilities, the leading mechanism is the electric dipole–dipole type, which shows variation of the energy transfer rate with the D–A distance (*d*) as

6 $$W_{DA}=\frac{1}{\tau_{D}}{\left(d_{0}/d\right)}^{6}$$

where τ_D_ is the radiative lifetime of the donor in the absence of the acceptor and *d*_0_ is the critical distance (excitation transfer and spontaneous deactivation of donor with equal probability). Herein, *d*_0_ follows the following expression

7 $$d_{0}^{6}=\frac{3}{64\pi^{5}}\frac{\sigma_{A}n_{D}^{0}}{\varepsilon^{2}{\mathrm{\nu ̅}}^{4}}\int_{0}^{\infty }g_{D}\left(\nu \right)g_{A}\left(\nu \right)\;d\nu$$

where ν is the wavenumber, ε the refractive index, *n*_D_^0^ is the quantum efficiency of the donor in the absence of the acceptor, ν̅ is the average wavenumber of the transition, σ_A_ the integrated absorption cross-section of the acceptor, and ∫*g*_D_(ν)*g*_A_(ν) dν represents the spectral overlap between donor emission and acceptor absorption.^43,44^ In phosphate glasses, it has been indicated that the energy transfer from Mn^2+^ ions to activators such as praseodymium^45^ and neodymium^46^ occurs mainly through the dipole–dipole interaction. Thus, even though not elucidated herein, an energy transfer from Mn^2+^ to Mn^3+^ ions taking place through such mechanism is conceivable. Overall, based on these considerations it may be suggested that besides the decrease in Mn^2+^ content implied by eq 1, the quenching pathway by Mn^2+^ → Mn^3+^ energy transfer is enhanced following γ-ray exposure as supported by the spectral overlap (Figure 10). In the presence of a nonradiative energy transfer, the lifetime of the energy donor decreases in the presence of the acceptor. Henceforth, the emission decay dynamics are evaluated seeking additional insights on the PL behavior of the glasses considered.

**Figure 10:**
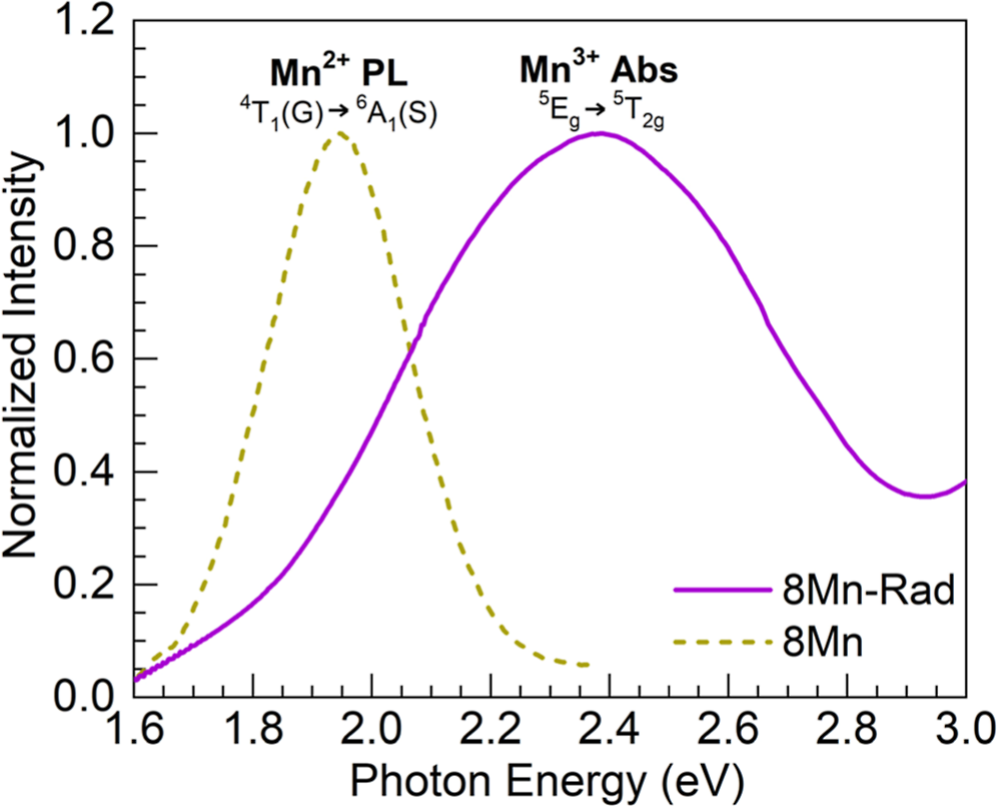
Spectral overlay of Mn^3+^ color center absorption in the γ-irradiated 8Mn-Rad glass with the emission from Mn^2+^ ions in the pristine 8Mn glass (λ_exc_ = 410 nm).

### Excited-State Dynamics Assessment

Emission decay curves were obtained for the pristine and γ-irradiated glasses under Mn^2+^ excitation at 410 nm by monitoring the emission from the ^4^T_1_(G) → ^6^A_1_(S) transition. Figure 11a,b show the semilog plots of the decay curves recorded under the conditions specified. The decays exhibit the characteristic behavior of Mn^2+^ ions, which tends to be multiexponential in nature with relatively slow decay times.^11,15,23,30,32^ The experimental curves were then fit within the typical framework of a biexponential decay as

8 $$I\left(t\right)=A_{f}\;\exp\left(\frac{-t}{\tau_{f}}\right)+A_{s}\;\exp\left(\frac{-t}{\tau_{s}}\right)$$

where *I*(*t*) is the time-dependent emission intensity, *A*_f_ and *A*_s_ are pre-exponential weight factors, and τ_f_ and τ_s_ are fast and slow lifetimes of Mn^2+^ ions taken as being interacting (e.g., Mn^2+^–Mn^2+^ pairs) or isolated Mn^2+^ ions, respectively.^11,15,30,47^ The solid traces shown over the data points in Figure 11a,b are the resulting fits (*R* ^2^ ≥ 0.96). The lifetime values estimated for each pristine and γ-irradiated glass sample are presented in Tables 4 and 5, respectively, along with the errors stemming from the fits. Noticing the results for the pristine 05–8Mn glasses in Table 4, it is seen that both τ_f_ and τ_s_ components tend to decrease. Decay time averages, τ_Ave_, may be further calculated^30,47^ from the fitting-deduced parameters through the following formula

9 $$\tau_{Ave}=\frac{A_{f}\tau_{f}^{2}+A_{s}\tau_{s}^{2}}{A_{f}\tau_{f}+A_{s}\tau_{s}}$$

The lifetime averages obtained for the pristine samples, τ_Ave,Pristine_, are 18.18, 18.05, 17.37, 16.73, and 15.53 ms, corresponding to 05Mn, 2Mn, 4Mn, 6Mn and 8Mn, respectively. Shorter lifetimes are expected for increasing Mn^2+^ concentrations in the glasses favoring a nonradiative depopulation via ion–ion interactions.^11,15,32^ Considering that the 05Mn and 2Mn glasses show decreased lifetimes while these glasses did not exhibit the prominent Mn^3+^ absorption in Figure 2a, it can be pointed that the energy transfer in these samples is largely between Mn^2+^ ions. However, the 4–8Mn glasses showed distinctively in Figure 2a the ^5^E_g_ → ^5^T_2g_ absorption characteristic of Mn^3+^ ions in octahedral coordination.^2,7,15,30^ Accordingly, it is likely that these participate as energy acceptors depopulating the ^4^T_1_(G) emitting level in both single and interacting Mn^2+^ ions. Observing the τ_f_ and τ_s_ values in Table 4, it appears that more drastic reductions occur for the fast lifetime component beyond the 4Mn glass (for a graphical appraisal see Figure 12a,b further discussed below). This suggests that Mn^2+^–Mn^2+^ pairs are at the origin of the PL quenching effect indicated to be manifested in Figure 6a for 6Mn and 8Mn. In such case, an additional quenching pathway involving such may be provided by the coexisting Mn^3+^ ions acting as energy sinks. In addition, the population of Mn^2+^ ions with fast decay time, *N*_f_, may be further estimated^30^ from the parameters in Table 4 through the following expression

10 $$N_{f}=\frac{A_{f}\tau_{f}}{A_{f}\tau_{f}+A_{s}\tau_{s}}$$

The resulting *N*_f_ percentages for the 05Mn, 2Mn, 4Mn, 6Mn and 8Mn glasses are 7.4%, 7.6%, 9.0%, 9.2%, and 10.1%, respectively, with the remainder percent being the population of ions with slow decay time. The increasing *N*_f_ trend indicates a greater concentration of interacting Mn^2+^ ions such as pairs participating in nonradiative energy transfer. This could be related to Mn^2+^–Mn^2+^ energy migration and ultimately the Mn^2+^ → Mn^3+^ energy transfer.

**Figure 11:**
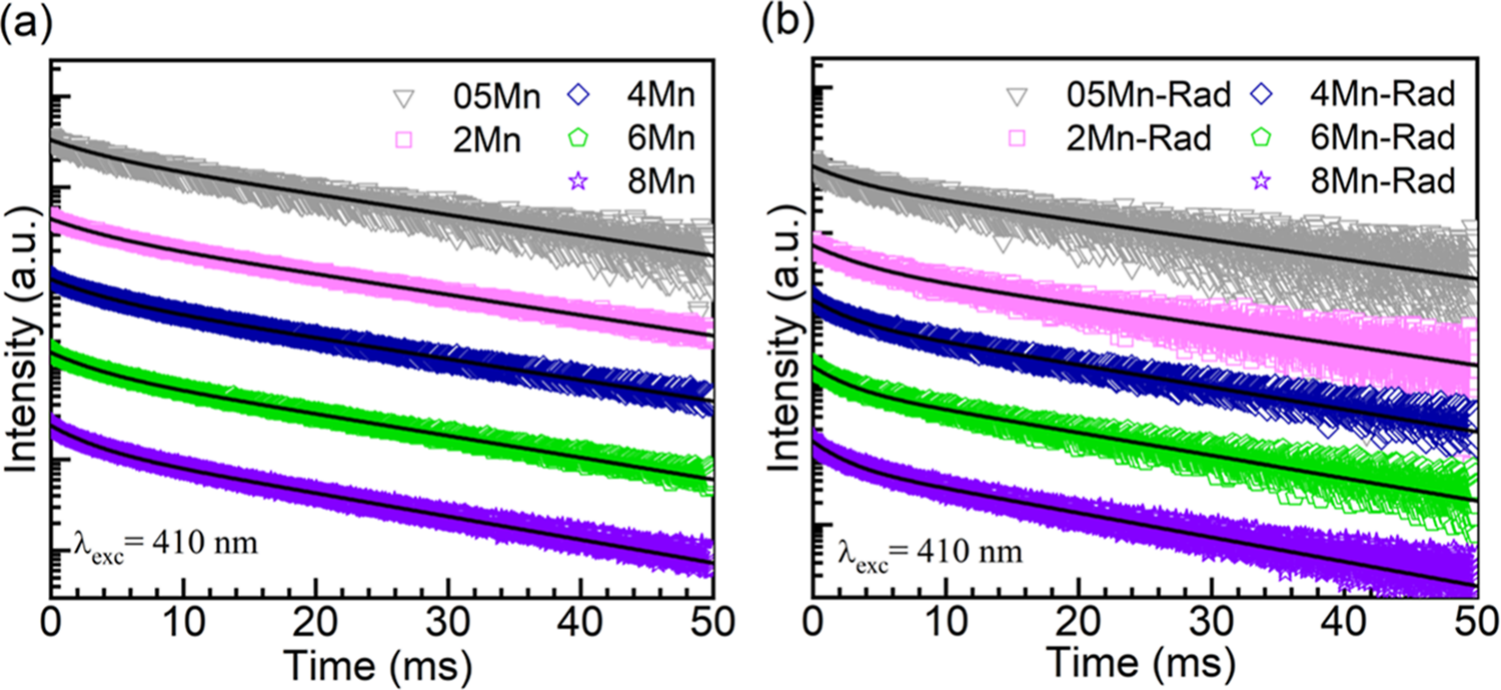
Semilog plots of emission decay curves for: (a) the pristine Mn-doped glasses and (b) the γ-irradiated counterparts. The solid lines are biexponential fits to the data (deduced parameters in Tables 4 and 5). The decay curves were all recorded under 410 nm excitation, while the detection wavelength was set at 615 nm for 05Mn and 05Mn-Rad, 620 nm for 2Mn and 2Mn-Rad, 630 nm for 4Mn and 4Mn-Rad, 638 nm for 6Mn and 6Mn-Rad, and 638 nm for 8Mn and 8Mn-Rad.

**Table 4:** Fast (τ_f_) and Slow (τ_s_) Lifetimes with Corresponding Weight Factors (*A*_f_, *A*_s_) Estimated for the Pristine Mn-Doped Glasses from the Experimental Decay Curves in Figure 11a

glass	*A*_f_	τ_f_ (μs)	*A*_s_	τ_s_ (μs)
05Mn	0.251 (±0.001)	3715 (±26)	0.606 (±0.001)	19,336 (±25)
2Mn	0.272 (±0.004)	3647 (±9)	0.631 (±0.004)	19,228 (±9)
4Mn	0.317 (±0.003)	3579 (±7)	0.609 (±0.004)	18,737 (±8)
6Mn	0.329 (±0.004)	3198 (±6)	0.573 (±0.004)	18,099 (±9)
8Mn	0.372 (±0.003)	2748 (±5)	0.535 (±0.004)	16,968 (±9)

**Table 5:** Fast (τ_f_) and Slow (τ_s_) Lifetimes with Corresponding Weight Factors (*A*_f_, *A*_s_) Estimated for the γ-Irradiated Mn-Doped Glasses from the Experimental Decay Curves in Figure 11b

glass	*A*_f_	τ_f_ (μs)	*A*_s_	τ_s_ (μs)
05Mn-Rad	0.288 (±0.001)	2688 (±25)	0.451 (±0.001)	16,327 (±40)
2Mn-Rad	0.371 (±0.001)	2911 (±15)	0.446 (±0.001)	15,687 (±29)
4Mn-Rad	0.409 (±0.005)	2260 (±5)	0.457 (±0.005)	14,190 (±11)
6Mn-Rad	0.417 (±0.007)	2142 (±6)	0.447 (±0.006)	13,970 (±15)
8Mn-Rad	0.435 (±0.005)	1954 (±5)	0.420 (±0.005)	13,005 (±12)

**Figure 12:**
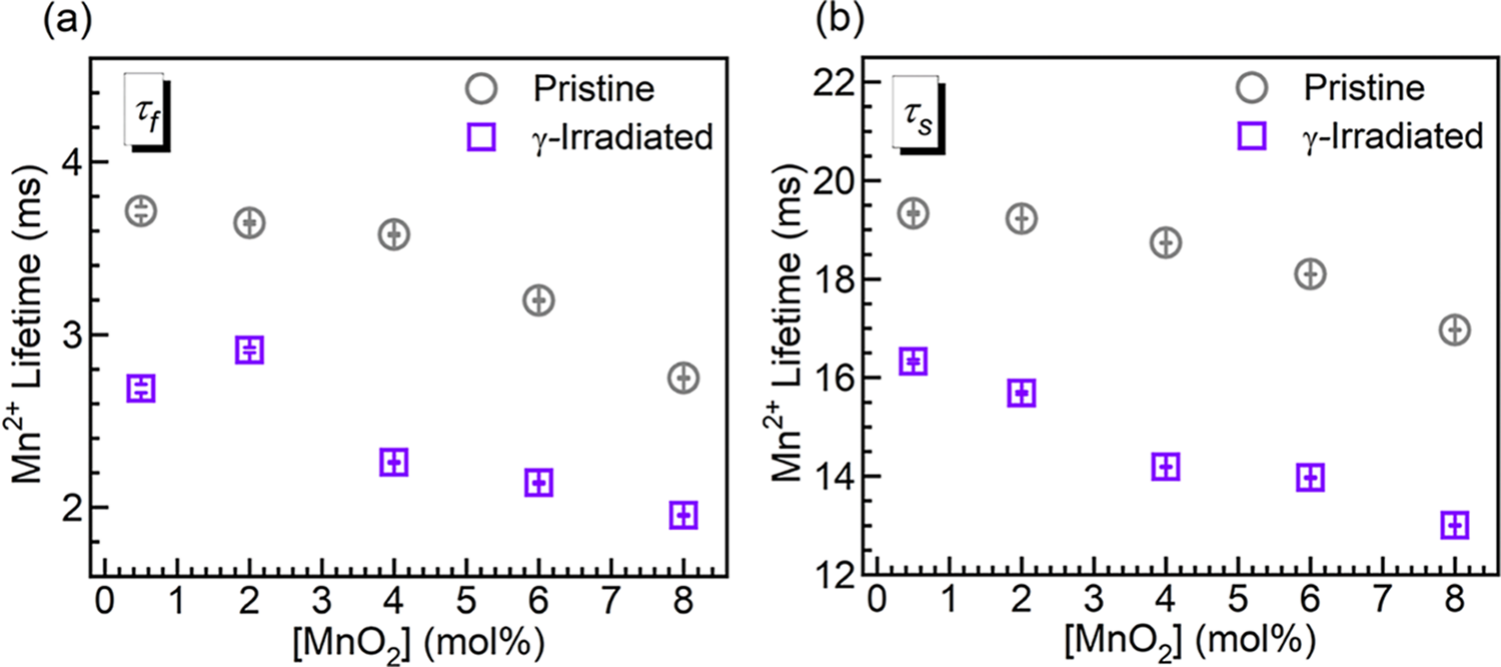
Plots of decay times of Mn^2+^ ions as a function of MnO_2_ added to the glasses (Tables 4 and 5) comparing the pristine with the γ-irradiated counterparts: (a) fast lifetime (τ_f_) component; and (b) slow lifetime (τ_s_) component.

The γ-irradiated glasses then show in Table 5 much shortened lifetimes compared to their pristine counterparts. Shown in Figure 12a,b are plots of the estimated τ_f_ and τ_s_ lifetimes for the pristine and γ-irradiated glasses as a function of MnO_2_ added to the glasses for visualization. The two sample sets exhibit in general a trend toward shorter Mn^2+^ decay time components, wherein the lifetimes for the γ-irradiated glasses are significantly shorter than the pristine counterparts. Decay time averages calculated through eq 9 from the fitting-deduced parameters in Table 5, τ_Ave,γ‑Irradiated_, are also shorter for the 05Mn-Rad, 2Mn-Rad, 4Mn-Rad, 6Mn-Rad, and 8Mn-Rad glasses, which decreased consistently as 15.03, 13.98, 12.70, 12.49, and 11.52 ms, respectively. Based on the decay times assessment, an estimate of the energy transfer rates may be obtained by subtracting the reciprocal of the Mn^2+^ average lifetime as the decay rate for the sample without the energy acceptor from that of the sample in which the transfer occurs.^47^ Hence, an elementary attempt is herein made to estimate empirically the quenching rate, *W*_Q_, following γ-irradiation through an equivalent expression as

11 $$W_{Q}=\frac{1}{\tau_{Ave,\gamma ‐Irradiated}}-\frac{1}{\tau_{Ave,Pristine}}$$

where τ_Ave,Pristine_ and τ_Ave,γ‑Irradiated_ are the average decay times of Mn^2+^ ions in the glasses before and after γ-irradiation, respectively, calculated from eq 9. Based on this basic approach, the *W*_Q_ values obtained in relation to the 05Mn-Rad, 2Mn-Rad, 4Mn-Rad, 6Mn-Rad, and 8Mn-Rad glasses were 11.54, 16.14, 21.15, 20.28, and 22.43 s^–1^, respectively. The *W*_Q_ values estimated are plotted in Figure 13 as a function of MnO_2_ content added to the glasses for a graphical appraisal. The quenching rates as calculated increase for the glasses containing 0.5–4 mol % MnO_2_ and fluctuate for 6 and 8 mol % MnO_2_, even though the latter exhibits the highest value. The low 0.5–4 mol % MnO_2_ concentration range shows strong linear correlation for the quenching rate as perceived from the regression analysis performed in Figure 13, exhibiting *r* and *R* ^2^ values of 0.998 and 0.997, respectively. It is likely that the departure from a continuous increase in *W*_Q_ at high MnO_2_ concentration is a reflection of the lifetime shortening already seen in the pristine glasses wherein Mn^3+^ absorption was quite significant.

**Figure 13:**
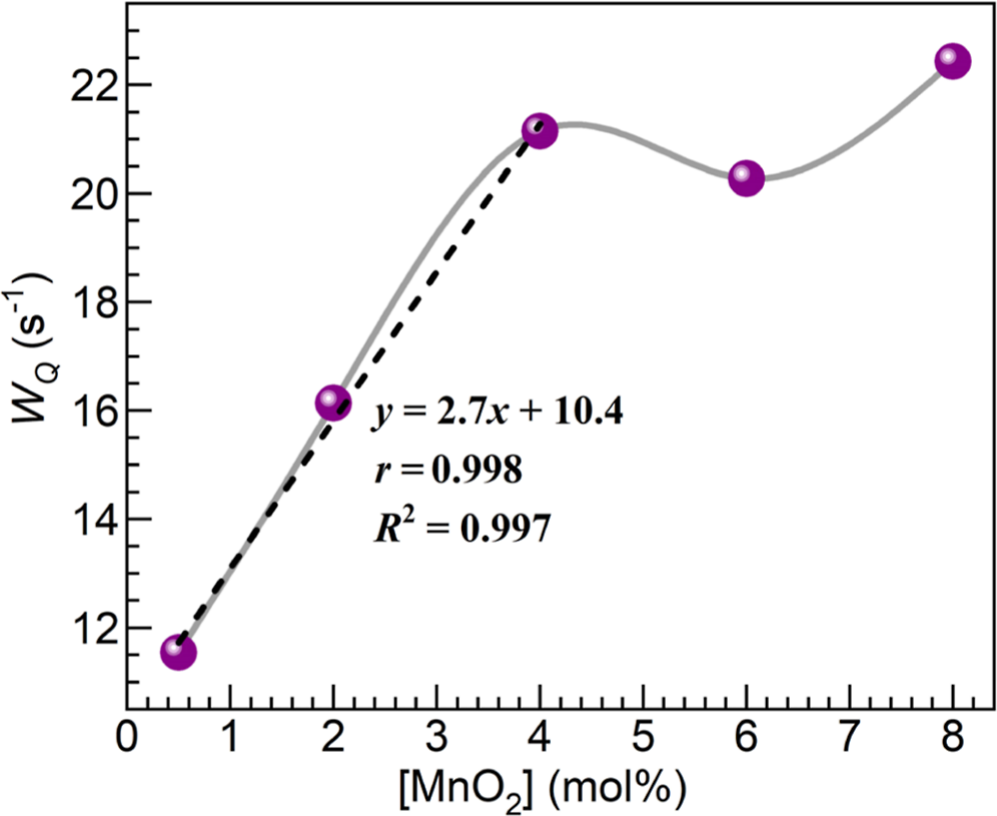
Plot of the empirical quenching rates following γ-irradiation (*W*_Q_) vs MnO_2_ content added to the glasses (the solid line is a guide to the eye); the dashed trace is a linear fit to the data for glasses made with 0.5–4 mol % MnO_2_ (equation, correlation coefficient, *r*, and coefficient of determination, *R* ^2^, displayed).

Concerning the origin of the lifetime shortening effect, it should be considered that the γ-irradiated glasses are expected to contain decreased Mn^2+^ contents^12^ in accord with their photo-oxidation represented by eq 1. In the absence of an additional quenching route, such a situation implies that the lifetimes of Mn^2+^ centers would increase as their concentration decreases, impeding Mn^2+^–Mn^2+^ interactions. This is for instance noticed in Table 4 when going down in concentration from the 2Mn pristine glass to the dilute 05Mn with the longest decay times, both of which lacked strong Mn^3+^ absorption in Figure 2a. Hence, the reduction in the lifetime values after γ-ray exposure supports the hypothesis of the PL quenching, Figure 8a–e, involving the energy transfer from the remaining Mn^2+^ ions to the photogenerated Mn^3+^ color centers. Further, through eq 10 and the parameters in Table 5, the population of Mn^2+^ ions with fast decay time, *N*_f_, for the 05Mn-Rad, 2Mn-Rad, 4Mn-Rad, 6Mn-Rad, and 8Mn-Rad glass samples were estimated at 9.5%, 13.4%, 12.5%, 12.5%, and 13.5%, respectively. The 05Mn-Rad glass exhibits the lowest percentage, yet the values fluctuate for the glasses with higher MnO_2_ content. Thus, unlike the pristine glasses, a sustained increasing trend in *N*_f_ is not observed after γ-irradiation. This may be related to the lowering in the Mn^2+^ concentration after γ-irradiation precluding Mn^2+^–Mn^2+^ interactions. However, compared to the pristine counterparts, *N*_f_ increments Δ*N*_f_ = *N*_f,γ‑Irradiated_ – *N*_f,Pristine_, after γ-irradiation of 2.1%, 5.8%, 3.4%, 3.3%, and 3.3%, were obtained for the 05Mn-Rad, 2Mn-Rad, 4Mn-Rad, 6Mn-Rad, and 8Mn-Rad samples, correspondingly. The increase in the population of interacting Mn^2+^ ions following γ-irradiation which decreases their concentration thus points to their involvement in Mn^2+^ → Mn^3+^ energy transfer.

The overall Mn^2+^ → Mn^3+^ transfer process can be finally represented in the schematic diagram shown in Figure 14, which illustrates the quenching of Mn^2+^ red emission via energy transfer to Mn^3+^ ions acting as energy acceptors. The Mn^2+^ ions are represented as being excited under at 410 nm, promoting ^6^A_1_(S) → ^4^E­(G),^4^A_1_(G) transitions. The excited Mn^2+^ ions subsequently decay nonradiatively to populate the emitting ^4^T_1_(G) level in the visible in resonance with the ^5^E_g_ → ^5^T_2g_ absorption in Mn^3+^ ions. The excited Mn^2+^ ions may decay radiatively via the ^4^T_1_(G) → ^6^A_1_(S) transition, or else the energy can be transferred resonantly to the ^5^T_2g_ state in Mn^3+^ ions. The Mn^3+^ energy acceptors then undergo nonradiative relaxation to the ^5^E_g_ ground state. This overall process illustrates the energy transfer pathway resulting in the PL quenching of the red emission from Mn^2+^ ions by the Mn^3+^ ions acting as energy sinks, the expression of which becomes enhanced by the γ-irradiation.

**Figure 14:**
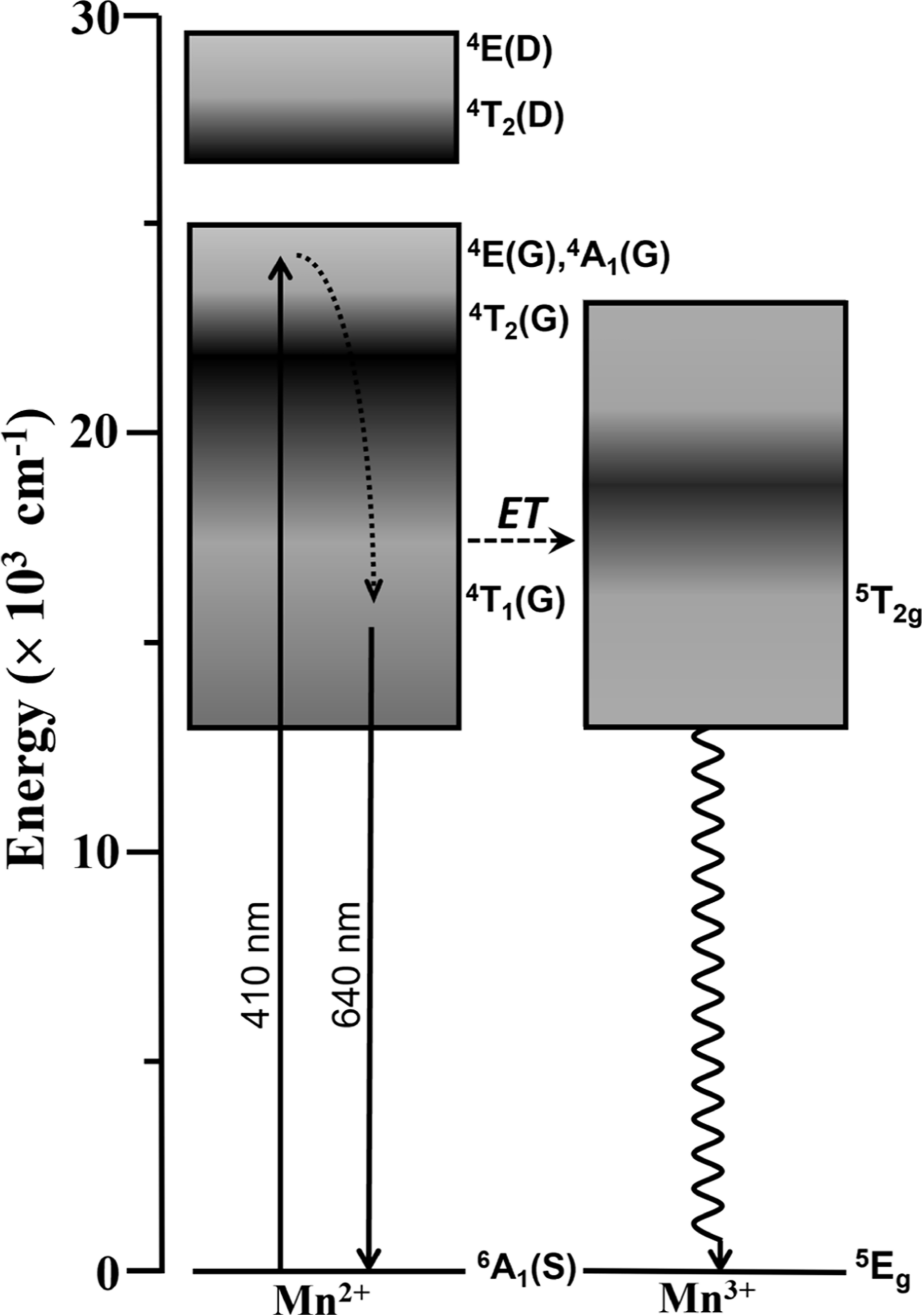
Simplified schematic illustrating energy transfer (ET) from red-emitting Mn^2+^ to Mn^3+^ as a PL quencher. The solid-straight arrows indicate radiative transitions in Mn^2+^ ions (i.e., direct excitation, spontaneous emission); the curved dotted arrow represents nonradiative feeding of the emitting level. The dashed horizontal arrow depicts the Mn^2+^ → Mn^3+^ resonant transfer and the solid curved arrow the nonradiative relaxation in Mn^3+^ ions acting as energy sinks.

## Conclusions

To summarize, the present investigation dealt with the influence of γ-irradiation (10 Mrad) on some physical and spectroscopic properties of barium phosphate glasses doped with MnO_2_ as 0.5, 2.0, 4.0, 6.0, and 8.0 mol % relative to P_2_O_5_. An evaluation of structural aspects by FT-IR spectroscopy harmonized in general with the preservation of the glass networks upon γ-irradiation. Nevertheless, the density appraisal showed that the more heavily doped glasses (2–8 mol % MnO_2_) were slightly denser after γ-irradiation. Given the lack of evidence in support of significant structural alteration, it was hypothesized that the increased densities reflected a compacting effect arising from strong Coulombic attraction between radiation-induced holes and electron centers. The absorption spectra for the pristine glasses presented distinct development of the ^5^E_g_ → ^5^T_2g_ transitions band characteristic of Mn^3+^ at high manganese content above 2 mol % MnO_2_. The optical band gap energies estimated were observed to decrease with increasing manganese doping likely due to O^2–^ → Mn^2+^ and O^2–^ → Mn^3+^ charge transfer transitions at high MnO_2_ concentrations. The γ-irradiated glasses all developed a deep purple hue and showed an intense absorption band centered within 520–540 nm. This was deemed indicative of the upsurge of radiation-induced Mn^3+^ color centers (also referred to as (Mn^2+^)^+^) due to photo-oxidation of Mn^2+^. The bandgap energies estimated for γ-irradiated glasses were all lower than the pristine counterparts. This was attributed to the occurrence of trapped electron centers in the glass matrix produced by γ-irradiation. All the pristine glasses exhibited the PL characteristic of Mn^2+^ ions, wherein the intensity evolution for the red ^4^T_1_(G) → ^6^A_1_(S) emission showed suppression above 4 mol % MnO_2_, indicating concentration quenching. In the γ-irradiated glasses, the PL from Mn^2+^ ions showed distinct evolution as the intensities dropped significantly above 4 mol % MnO_2_. Further, the red ^4^T_1_(G) → ^6^A_1_(S) emission was always quenched in the γ-irradiated glasses relative to the pristine versions. The CIE 1931 chromaticity coordinates and CCT evaluation then showed that the γ-irradiated samples exhibited a shift from red toward a more orange character and higher color temperatures at low manganese content. Comparison of the integrated PL intensities before and after γ-irradiation exposed that the emission quenching became more pronounced with increasing manganese content likely associated with photogenerated Mn^3+^ color centers. The decay kinetics evaluation for the pristine glasses showed fast (e.g., Mn^2+^–Mn^2+^ dimers) and slow (single Mn^2+^ ions) lifetime components decreasing throughout the entire glass set. Besides Mn^2+^–Mn^2+^ interactions, a PL quenching via Mn^2+^ → Mn^3+^ energy transfer was suggested, the prominence of which increased above 4 mol % MnO_2_. Estimates for the population of fast-decaying Mn^2+^ ions showed their increased involvement in energy transfer as the MnO_2_ content increased in the pristine glasses. The fast and slow lifetime components of Mn^2+^ ions after γ-irradiation were then all shorter than the pristine counterparts. Herein then, besides the decreased Mn^2+^ content after γ-irradiation likely causing weaker emission, a resonant energy transfer from Mn^2+^ to the photogenerated Mn^3+^ was proposed as a significant pathway leading to PL quenching. The population of fast-decaying Mn^2+^ ions was then always found to be higher in the γ-irradiated samples than the pristine. Thus, their involvement in Mn^2+^ → Mn^3+^ energy transfer was suggested to take place while the Mn^2+^ concentration decreased upon γ-irradiation. An empirical appraisal of the PL quenching rate was made based on the average decay times, wherein the low 0.5–4 mol % MnO_2_ concentration range showed a strong linear correlation with MnO_2_ content. Departure from linearity was seen afterward, likely reflecting the lifetime shortening already seen in the pristine glasses wherein Mn^3+^ absorption was quite significant. The overall steady-state and kinetics assessment thus bears important physical insights allowing for the conclusion that Mn^2+^ → Mn^3+^ resonance energy transfer is a dominant emission quenching pathway for systems with significant Mn^3+^ content irrespective of their processing. Still, additional fundamental work is desired to elucidate the nature of the energy transfer mechanism (e.g., electric dipole–dipole vs dipole–quadrupole, etc.). Results ratify that for light-emitting applications Mn^3+^ impurities should be reduced as much as possible to avoid limiting device optical output. The effect of γ-irradiation is therefore detrimental to such aspect as Mn^2+^ is photo-oxidized; however it remains relevant to other areas such as optical dosimetry and photochromic lenses.

## Supplementary Material



## Notes

Deconvolution results for the FT-IR spectra of the different glasses studied (PDF)
