# Supplementary material for: Optical Interactions and Excited-State Dynamics in γ‑Irradiated Mn-Doped Phosphate Glasses
Source: J Phys Chem B. 2026 Jun 25;130(27):7008–21. doi: 10.1021/acs.jpcb.6c02415 (PMC13359106; doi:10.1021/acs.jpcb.6c02415)
Supplement: Supplementary file 1 [file jp6c02415_si_001.pdf]

Supporting Information

Optical Interactions and Excited-State Dynamics in γ-Irradiated Mn-Doped Phosphate Glasses

José A. Jiménez *

Center for Advanced Materials Science, Department of Biochemistry, Chemistry & Physics, Georgia Southern University, Statesboro, GA 30460, USA

*E-mail: jjimenez@georgiasouthern.edu

ORCID:

José A. Jiménez: **0000-0001-9256-3836**

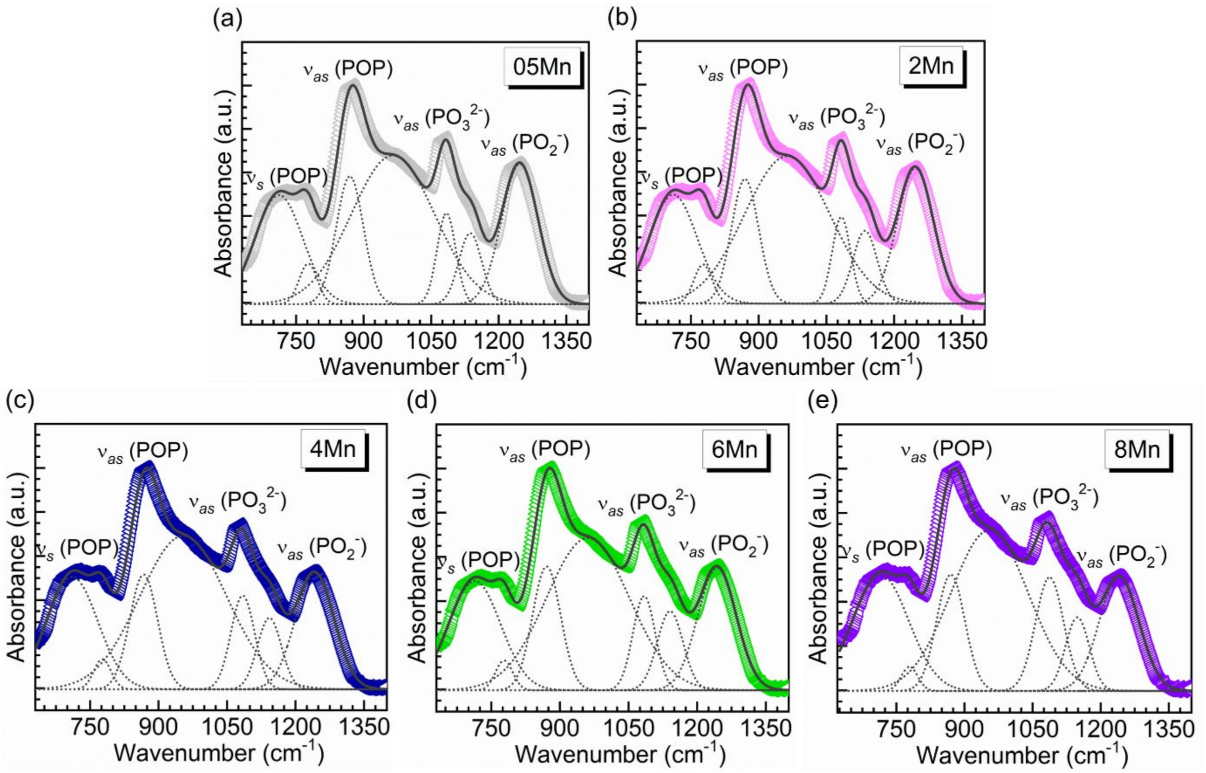

Fig. S1. FT-IR spectra deconvolution results (deduced parameters in Table S1) for the pristine Mn-doped phosphate glasses (the experimental data points are the colored symbols; the cumulative fits are the solid traces within the symbols): (a) 05Mn; (b) 2Mn; (c) 4Mn; (d) 6Mn; and (e) 8Mn.

Table S1. Band positions, full-width at half-maximum (FWHM) values, and assignments for the different features of the pristine Mn-doped phosphate glasses resulting from the FT-IR spectral deconvolutions (Fig. S1).

	Band vibrational frequency (FWHM) / cm^{-1}				
Assignments	05Mn	2Mn	4Mn	6Mn	8Mn
Symmetric P–O–P stretch due to pyrophosphate groups	712 (124)	711 (124)	711 (132)	714 (133)	715 (146)
Symmetric P–O–P stretching in metaphosphate chains	777 (48)	777 (48)	777 (44)	778 (44)	779 (43)
Asymmetric P–O–P stretch in metaphosphate chains	869 (71)	870 (71)	867 (72)	871 (72)	870 (73)
Asymmetric P–O–P stretch of metaphosphate groups in various size rings	965 (218)	963 (222)	954 (224)	961 (214)	955 (201)
Asymmetric stretch from PO_3^{2-} groups	1084 (49)	1083 (48)	1084 (61)	1084 (58)	1087 (73)
Symmetric stretch from PO_2^{-} groups	1137 (60)	1136 (63)	1143 (62)	1141 (67)	1149 (62)
Asymmetric stretch from PO_2^{-} groups	1247 (96)	1247 (95)	1242 (100)	1243 (99)	1241 (103)

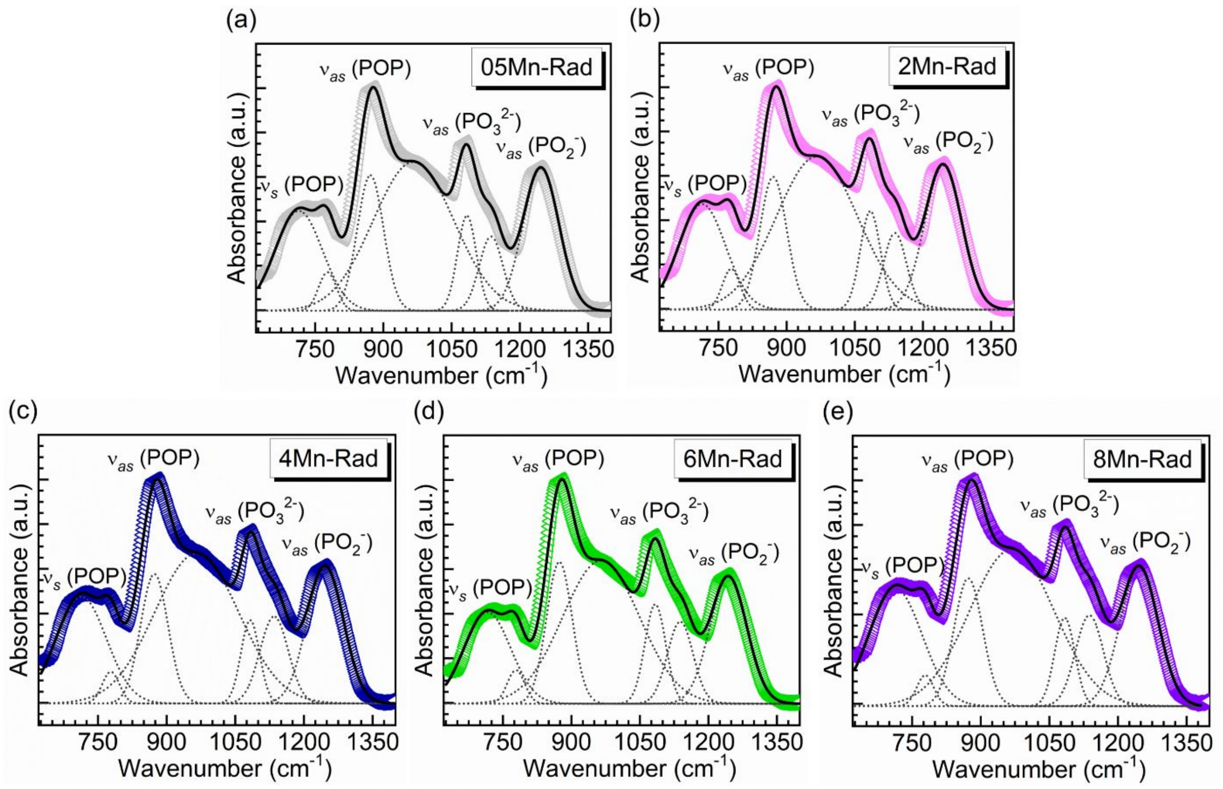

Fig. S2. FT-IR spectra deconvolution results (deduced parameters in Table S2) for the γ-irradiated Mn-doped phosphate glasses (the experimental data points are the colored symbols; the cumulative fits are the solid traces within the symbols): (a) 05Mn-Rad; (b) 2Mn-Rad; (c) 4Mn-Rad; (d) 6Mn-Rad; and (e) 8Mn-Rad.

Table S2. Band positions, full-width at half-maximum (FWHM) values, and assignments for the different features of the γ-irradiated Mn-doped phosphate glasses resulting from the FT-IR spectral deconvolutions (Fig. S2).

	Band vibrational frequency (FWHM) / cm^{-1}				
Assignments	05Mn-Rad	2Mn-Rad	4Mn-Rad	6Mn-Rad	8Mn-Rad
Symmetric P–O–P stretch due to pyrophosphate groups	714 (123)	714 (121)	713 (130)	716 (120)	714 (137)
Symmetric P–O–P stretching in metaphosphate chains	777 (46)	778 (46)	778 (44)	778 (45)	779 (44)
Asymmetric P–O–P stretch in metaphosphate chains	872 (68)	870 (70)	873 (70)	873 (66)	873 (74)
Asymmetric P–O–P stretch of metaphosphate groups in various size rings	966 (209)	967 (211)	966 (217)	965 (199)	968 (218)
Asymmetric stretch from PO_3^{2-} groups	1084 (49)	1084 (52)	1083 (48)	1084 (54)	1084 (52)
Symmetric stretch from PO_2^{-} groups	1137 (62)	1139 (60)	1135 (73)	1138 (66)	1138 (72)
Asymmetric stretch from PO_2^{-} groups	1246 (94)	1245 (96)	1247 (94)	1244 (95)	1246 (98)
